# Pravastatin Corrects Endothelial Dysfunction in Ex Vivo Uterine Radial Arteries in Preeclampsia

**DOI:** 10.1111/apha.70186

**Published:** 2026-03-11

**Authors:** Nathan M. Luque, Leo Leader, Sandra M. Lowe, Steven D. Horrowitz, Marianne Tare, Victoria Hinkley, Vladimir V. Matchkov, Maged M. Costantine, Irit Markus, Lu Liu, Shaun L. Sandow, Timothy V. Murphy

**Affiliations:** ^1^ Physiology, School of Biomedical Sciences University of New South Wales Sydney New South Wales Australia; ^2^ Medicine, Blacktown Clinical School and Research Centre Western Sydney University Blacktown New South Wales Australia; ^3^ Women's Health and Royal Hospital for Women University of New South Wales Sydney New South Wales Australia; ^4^ Physiology, and Monash Rural Health Monash University Warragul Victoria Australia; ^5^ Biomedical Science University of the Sunshine Coast Sippy Downs Queensland Australia; ^6^ UQ Centre for Clinical Research University of Queensland Herston Queensland Australia; ^7^ Department of Biomedicine Aarhus University Aarhus Denmark; ^8^ Maternal Fetal Medicine, Department of Obstetrics & Gynecology Ohio State University Wexner Medical Center Columbus Ohio USA; ^9^ Pharmacology University of New South Wales Sydney New South Wales Australia

**Keywords:** calcium‐activated potassium channels, caveolae, endothelium‐dependent dilation, gap junction, nitric oxide

## Abstract

**Aim:**

Endothelium‐dependent relaxation in isolated uterine radial arteries from normotensive (NT) and preeclamptic (PE) pregnancies, and the acute effects of pravastatin in the latter vessels were assessed. Pravastatin is hypothesized to alleviate endothelial dysfunction in PE via modulating aspects of NO and endothelium‐derived hyperpolarization‐mediated relaxation.

**Methods:**

Radial arteries isolated from the uterus of NT and PE pregnant patients were incubated with pravastatin (2 mM/6 h), methyl‐β‐cyclodextrin (10 mM/1 h) in vitro, or vehicle. Vessel function was determined with pressure myography, while related morphology and protein/mRNA expression were characterized using immunohistochemistry, electron microscopy, and qPCR.

**Results:**

Endothelium‐dependent, bradykinin‐induced NO‐mediated relaxation was impaired in radial arteries from PE compared to NT pregnancy, with a reduced intermediate‐ and large‐conductance Ca^2+^‐activated K^+^‐channel contribution. Endothelial small‐conductance Ca^2+^‐activated K^+^‐channel function and expression were increased in arteries from PE, compared to NT patients. Pravastatin restored NO and endothelium‐derived hyperpolarization‐mediated relaxation in arteries from PE women; potentially overcompensating overall endothelium‐dependent relaxation. Myoendothelial gap junction and endothelial caveolae density, and caveolin‐1 and endothelial‐NOS expression were decreased in arteries from PE relative to NT pregnancies and increased following pravastatin incubation. Caveolae density in NT patient arteries was reduced by methyl‐β‐cyclodextrin, while endothelial caveolae were increased in vessels from PE patients. Pravastatin incubation restored endothelial function via improved NO and endothelium‐derived hyperpolarization‐type mechanisms.

**Conclusions:**

Pravastatin restored endothelium‐dependent relaxation in uterine radial arteries from PE pregnancies. Data support the therapeutic potential for pravastatin in treating PE, with ongoing trials determining the validity of its use in the clinical setting.

**Trial Registration:**

ClinicalTrials.gov identifier: NCT01717586

**Practitioner Points**

- Uterine radial arteries isolated from preeclampsia patients show impaired endothelium‐dependent relaxation via loss of nitric oxide and large‐conductance Ca^2+^‐activated K^+^‐channel activity, with reduced anatomical myoendothelial gap junction coupling.
- Pravastatin restores endothelium‐dependent relaxation in these ex vivo uterine radial arteries from preeclamptic pregnancy, primarily through increased nitric oxide and endothelium‐derived hyperpolarization mechanisms.
- A potential mechanistic basis for pravastatin action in treating preeclampsia, where it may reduce hypertension and improve organ perfusion to correct the dysfunction in the disease state is demonstrated.

## 1 Introduction

Preeclampsia (PE) is a leading cause of maternal, fetal and neonatal morbidity and mortality [1], complicating 2%–8% of pregnancies worldwide [1]. Both short and long‐term cardiovascular risk are increased in PE [1], with altered uterine vascular resistance and dysfunctional endothelium‐dependent relaxation being key factors [2, 3]. Preeclampsia is associated with abnormal placentation and a failure of uterine vasculature adaptation, along with a range of downstream events [4, 5]. The management of PE is limited to treatments which are directed at controlling hypertension, coagulopathy and fluid derangement, with early planned delivery [6]. These approaches have no effects on the underlying pathophysiology and do not address the long‐term cardiovascular and metabolic disease which occurs more commonly after PE [7]. Therapies that can address the underlying microvascular dysfunction are needed for prevention and treatment of PE and reduction of these long‐term risks [7].

Although successful PE therapeutics remain elusive, pravastatin has been proposed as a preventative strategy in high‐risk patients, initiating treatment < 20 weeks gestation [8]. The INOVASIA Study [9] showed twice‐daily pravastatin in a group of 87 pregnant women at high risk of developing PE ultimately halved the rate of pre‐term PE and reduced pre‐term births by two‐thirds, compared to the control group [9]. Earlier meta‐analyses concluded pravastatin reduced both the rate of both PE and preterm births [10], or pre‐term births only [11]. Studies support the maternal safety profile of pravastatin in pregnancy [12, 13], with no differences in users and non‐users reporting general symptoms associated with pregnancy. Given the analyses in these studies, and the inherent limitations therein, further translational investigation is required. The pleiotropic effects of pravastatin treatment in patients with PE likely involve mechanisms improving vascular endothelial function [14, 15], independent of their statin action [16]. Notably, pravastatin lowers lipids via two main pathways, as a functional hydroxymethylglutaryl‐CoA reductase inhibitor, with secondary effects blocking LDL synthesis. Its additional non‐statin pleiotropic effect is the primary interest of its use as a PE treatment [8, 15, 16]. In comparison, other agents such as methyl‐β‐cyclodextrin (MβCD) exert more singular, cholesterol‐specific action without engaging intracellular signaling pathways, sequestering cholesterol directly from the plasma membrane via its hydrophobic core [17].

The endothelium‐dependent mechanisms which determine uterine radial artery blood pressure are not well established in normotensive (NT) or PE pregnancy. Mechanisms of endothelium‐dependent relaxation involve NO, prostacyclin, and endothelium‐derived hyperpolarization (EDH) of vascular smooth muscle, with the latter being dominant in small peripheral resistance arteries where blood pressure is primarily determined [18], including in the uterus [5, 18, 19, 20, 21]. Endothelium‐derived hyperpolarization may occur via several mechanisms, but ultimately depends upon the coordinated activation of calcium‐activated K^+^ channels (K_Ca_), with small and intermediate conductance (S/I)K_Ca_ generating hyperpolarizing currents in the endothelium which can spread to the smooth muscle via myoendothelial gap junctions [18, 22, 23]. While large (B)‐conductance K_Ca_ values are usually restricted to smooth muscle cells, they may be activated by chemical messengers derived from the endothelium [24, 25]. Notably, mechanisms of EDH have not been well defined in the microvasculature of the human uterus in normal healthy or PE pregnancies [26, 27]. Characterization of EDH in uterine arteries has been examined in animal models of hypertension in pregnancy; albeit, such models do not appropriately reflect PE physiology, as occurrence of this disease is limited to higher primates [28, 29, 30].

In vascular disease‐related signaling, the regulatory roles of endothelial cells are associated with angiogenic, inflammatory, and immune processes [4, 31, 32, 33]. These pathways are also linked to ubiquitous endothelial cell caveolae, cholesterol‐rich, omega‐shaped membranous and submembranous vesicle‐like structures where components of NO and EDH activity have suggested spatial localization in some vascular beds and states [31, 34, 35, 36, 37, 38]. The cholesterol‐dependent nature of caveolae renders them potential targets for pravastatin action [31, 32, 39, 40].

This study determines the mechanisms of endothelium‐dependent relaxation in ex vivo uterine radial arteries from control healthy NT and PE pregnancies, with a focus on EDH‐type and NO‐signaling. Uterine radial arteries are examined as the most distal resistance vessels in the pregnant uterus, and thus a primary site for determination of blood pressure in PE [41]. The effect and mechanism/s of acute pravastatin action in endothelium‐dependent relaxation were determined, clarifying the hypotheses that this agent ameliorates endothelial dysfunction in ex vivo uterine radial arteries in PE via altering aspects of EDH and NO‐related vascular signaling.

## 2 Results

### 2.1 Broad Protocol

In freshly isolated human uterine radial arteries from pregnant NT and PE patients, functional endothelium‐dependent relaxation mechanisms were determined using pressure myography with pharmacological intervention. The related distribution and expression of the primary endothelium‐dependent relaxation signaling proteins and their mRNA associated with EDH and NO dilator activity were determined using confocal and electron microscopy, immunohistochemistry, and qPCR.

### 2.2 Patients and Tissue

Tissue was available from 107 NT and 31 PE consenting patients with final inclusion based on sufficient artery retrieval from viable myometrial tissue collected at caesarean section, with NT samples appropriately matched to PE tissue availability. Patient characteristics show that maternal age was similar between the NT and PE groups; although patients with PE had higher body weight and body mass index upon first consultation, with increased systolic and diastolic blood pressures and lower gestational age. PE patients also presented with proteinuria (100%) and fetal growth restriction (50%), which were absent in NT (Table 1).

**TABLE 1:** Patient data from human pregnancies.

	Normotensive (*n* = 14)	Preeclamptic (*n* = 10)
Maternal age (years)	34.4 ± 0.9	34.4 ± 1.2
Weight at first consult (kg)	56 ± 2	79 ± 7*
Height (cm)	160 ± 2	167 ± 3*
Body mass index (weight, kg/height, m^2^)	21.6 ± 0.6	28.4 ± 2.2*
Systolic blood pressure (mmHg)	120 ± 0.4	159 ± 7*
Diastolic blood pressure (mmHg)	78 ± 2	100 ± 3*
Gestational age (days)	273 ± 2	229 ± 9*
Infant birth weight (g)	3319 ± 91	1573 ± 215*
% with proteinuria	0	100%
% with fetal growth restriction	0	50%
Gravida	2.0 ± 0.8	1.7 ± 0.3
Parity	0.9 ± 0.2	0.9 ± 0.3
Infant sex, male: female	7: 7	7: 4
Twin pregnancies	0	1 (F.F; as fraternal)

* Significant difference of preeclamptic from normotensive control (*p* < 0.05); unpaired two‐tailed *t*‐test; mean ± SEM. ‘*n*’, each from different patients.

### 2.3 Effect of PE, Pravastatin, and MβCD on Endothelial Function of Uterine Radial Arteries

**FIGURE 1:**
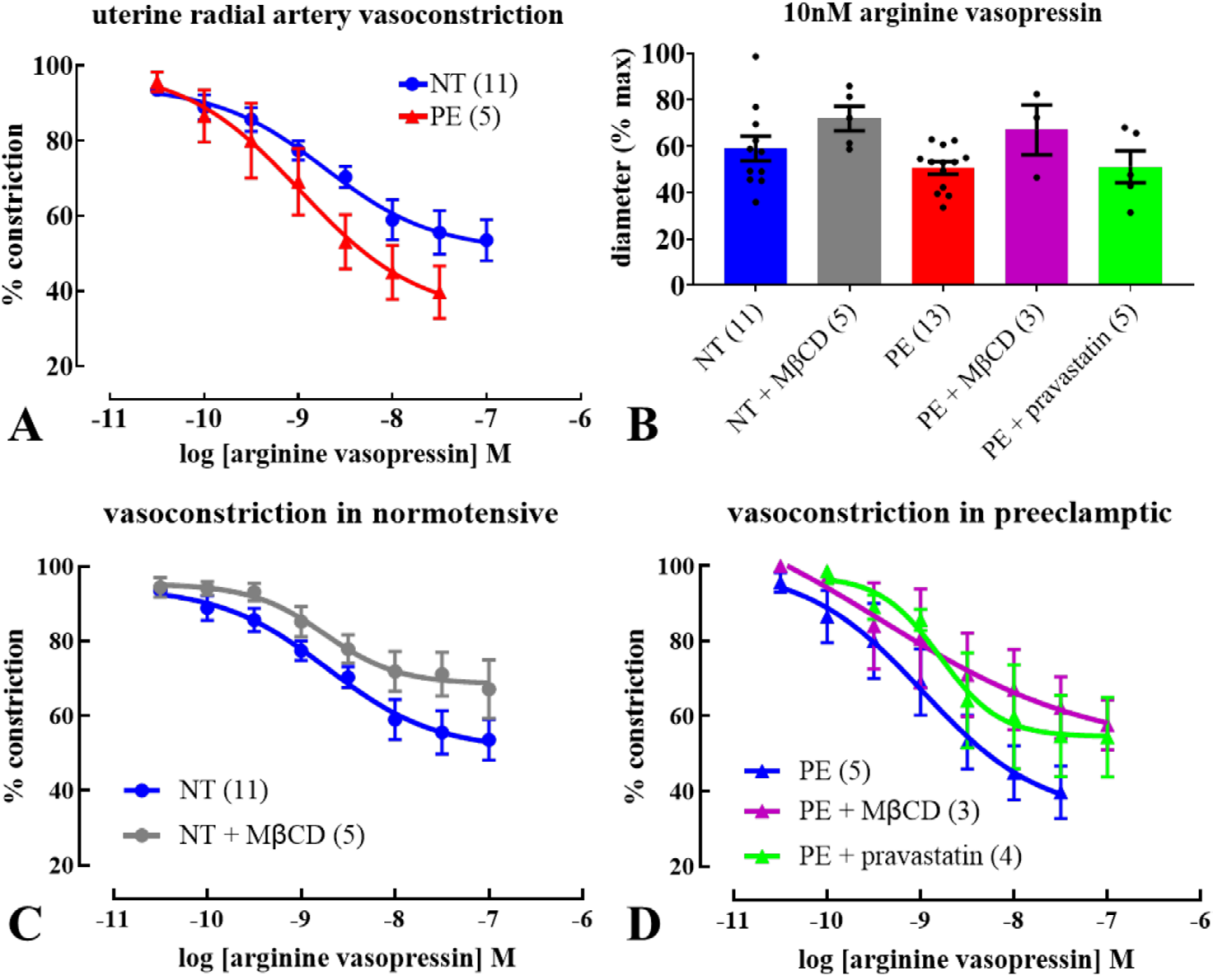
Vasopressin‐induced constriction in uterine radial arteries in pregnancy. Concentration‐constriction relationships for arginine vasopressin in isolated, pressurized (60 mmHg) uterine radial arteries from untreated normotensive (NT) and preeclamptic (PE) pregnancies (A), as well as comparison of responses to 10 nM arginine vasopressin (B). The effects of acute methyl‐β‐cyclodextrin (MβCD; 10 mM/1 h) or pravastatin (2 mM/6 h) incubation on concentration‐constriction relationships for arginine vasopressin from NT (C) and PE (D) are also shown. Symbols/columns represent mean ± SEM of diameter normalized to maximum (in zero [Ca^2+^]); ‘*n*’ in parentheses. Responses to arginine vasopressin were not significantly altered by any treatment or condition (*p* > 0.05, one‐way ANOVA with Šídák's post hoc multiple comparisons test where appropriate). See also Table S1.

To assess comparative endothelium‐dependent relaxation, matched baseline radial artery tone is induced via vasopressin pre‐constriction at 60 mmHg. Mean passive artery inner diameter was 172 ± 7 (*n* = 22) and 188 ± 21 μm (*n* = 11) in NT and PE, respectively (*p* > 0.05). The response to 10 nM and concentration‐dependent constriction to arginine vasopressin did not differ in arteries from PE compared with NT patients (Figure 1A,B; Table S1). Neither pravastatin nor MβCD (cholesterol depleting agent [17]) treatment significantly altered responses to arginine vasopressin in arteries from NT or PE pregnancies., M (Figure 1C,D; Table S2).

**FIGURE 2:**
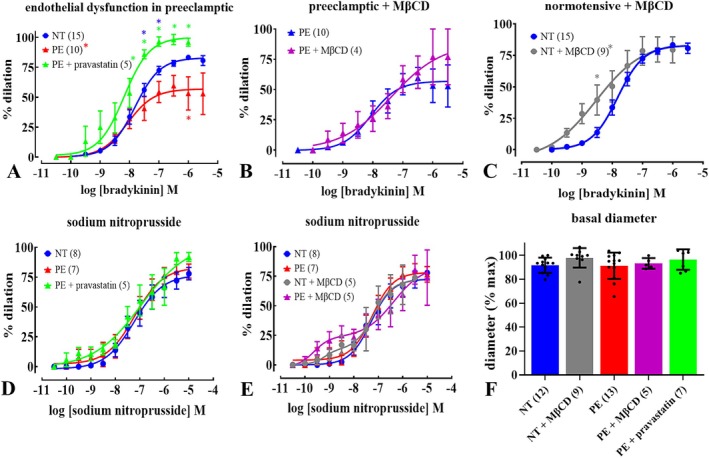
Pravastatin restores endothelium‐dependent relaxation in preeclampsia (PE). Concentration‐relaxation relationships for bradykinin (A–C) or sodium nitroprusside (D, E) in isolated, pressurized (60 mmHg) pre‐constricted (arginine vasopressin; 10 nM) uterine radial arteries from normotensive (NT) and PE pregnancies either as untreated or incubated with pravastatin (2 mM/6 h) or methyl‐β‐cyclodextrin (MβCD; 10 mM/1 h). Symbols represent mean ± SEM of percentage relaxation, ‘*n*’ in parentheses. For comparison of PE, PE + pravastatin and NT response to bradykinin (A): Red asterisks (*) indicate PE, as significantly different from NT; green asterisks (*) indicate PE + pravastatin as significantly different from PE; and blue asterisks (*) indicate PE + prav as significantly different from NT; *p* < 0.05, two‐way ANOVA with Šídák's post hoc multiple comparisons test. *Interaction effect as PE *cf/*. NT (red), PE + pravastatin *cf/*. PE (green). For MβCD (B, C) and sodium nitroprusside (D, E) *p* > 0.05, two‐way ANOVA with Šídák's post hoc multiple comparisons test; C, *p < 0.05. Basal diameter (F) in pressurized (60 mmHg) uterine radial arteries from NT and PE pregnancies for each treatment is additionally shown, each from different patients; columns represent mean ± SEM of diameter normalized to maximum (in zero [Ca^2+^]) *p* > 0.05, one‐way ANOVA. See also Table S2.

Initial studies examined vasodilation in response to pharmacological stimulation in arteries from women with NT or PE pregnancy, or PE with subsequent pravastatin incubation. Arteries from patients with PE had reduced bradykinin (endothelium‐dependent vasodilator [42, 43, 44])‐induced endothelium‐dependent relaxation compared to those from NT. Relative endothelium‐dependent relaxation in arteries from PE pregnancies increased following pravastatin incubation (Figure 2A; Table S3). Pravastatin reduces membrane cholesterol levels, so experiments in PE arteries were performed with another membrane cholesterol‐lowering agent, MβCD. Treatment with MβCD did not affect bradykinin‐induced dilation of arteries from PE women, but increased the sensitivity of vessels from NT patients to bradykinin (Figure 2B,C; Table S3). Endothelium‐independent relaxation of arteries induced by sodium nitroprusside did not differ between arteries from NT and PE pregnancies, suggesting endothelium‐dependent mechanisms of dilation were selectively inhibited in PE, as opposed to the general ability of smooth muscle to relax. Neither pravastatin nor MβCD altered sodium nitroprusside (NO donor [42, 43, 44])‐induced relaxation (Figure 2D,E; Table S3). Neither pravastatin nor MβCD altered resting baseline diameter of arteries from NT or PE pregnancies (Figure 2F; Table S3). Thus, endothelium‐dependent vasodilation of uterine arteries was inhibited in arteries from women with PE, relative to vessels from NT pregnancies, and this inhibition was reversed by pravastatin.

**FIGURE 3:**
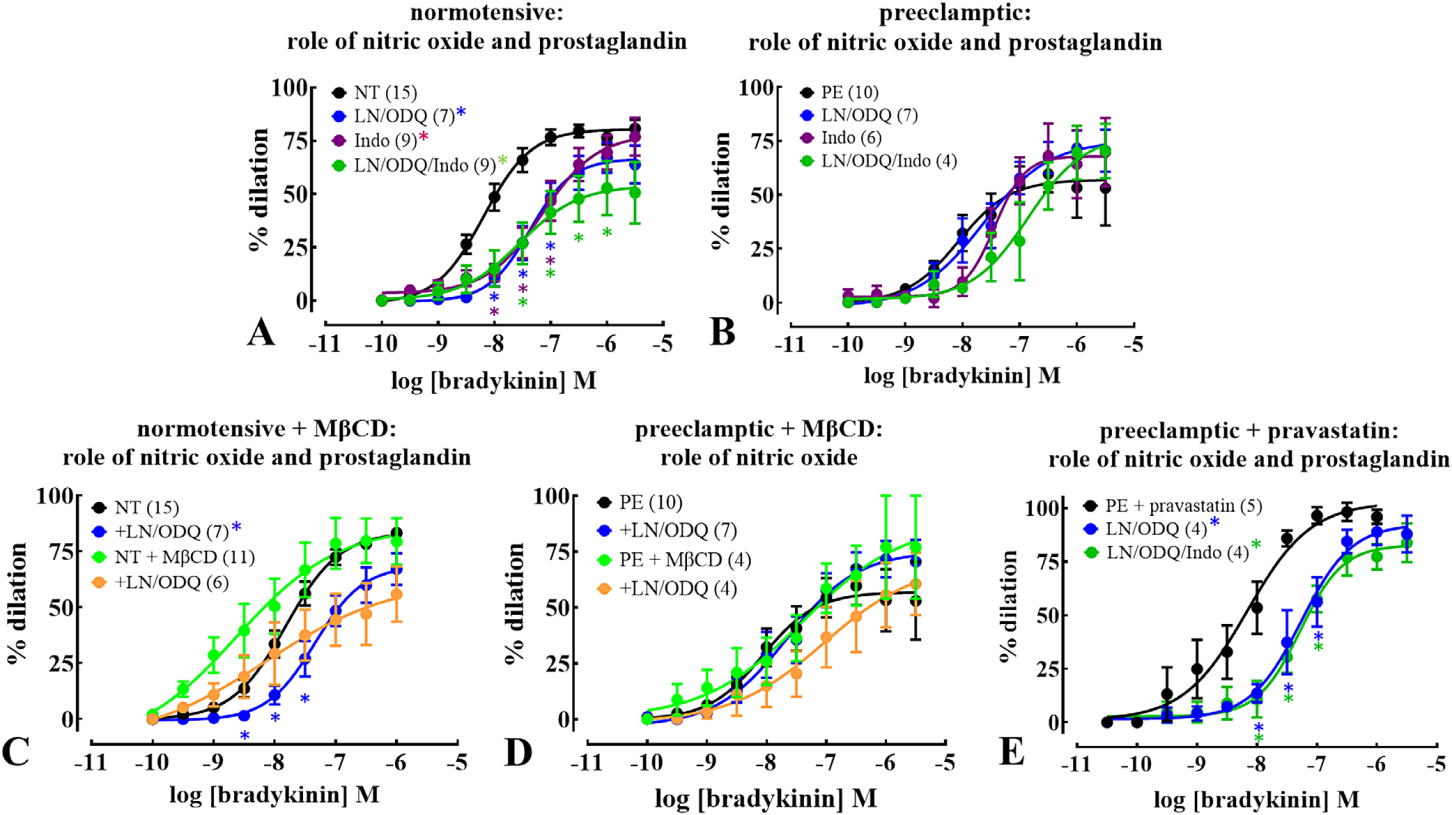
Pravastatin restores NO and prostaglandin‐mediated uterine radial artery relaxation in preeclampsia (PE). Concentration‐ relaxation relationships for bradykinin in isolated, pressurized (60 mmHg) pre‐constricted (arginine vasopressin; 10 nM) uterine radial arteries from normotensive (NT; A, C) and PE (B, D, E) pregnancies. Tissues examined were either untreated (A, B), incubated at 37°C in methyl‐β‐cyclodextrin (MβCD; cholesterol depleting agent; 10 mM; C, D) or in pravastatin (2 mM/6 h; E). Incubation with *N*_ω_‐nitro‐L‐arginine methyl ester HCl (L‐NAME; LN; 100 μM/30 min) and 1H‐[1,2,4]oxadiazolo[4,3‐a]quinoxalin‐1‐one (ODQ; 10 μM/30 min), and indomethacin (Indo; 10 μM/30 min) inhibited NOS, soluble guanylyl cyclase (sGC) and COX, respectively. Incubation with LN/ODQ reduced sensitivity to bradykinin‐induced relaxation in NT (A) but not PE (B) and significantly reduced sensitivity to bradykinin‐induced relaxation in pravastatin‐treated PE (E) arteries, denoted by a shift in pEC_50_. Indomethacin (Indo; 10 μM/30 min) inhibited bradykinin‐induced relaxation in uterine radial arteries from NT (A). LN/ODQ/Indo did not produce further inhibition of bradykinin‐induced relaxation in combination, but significantly reduced maximum relaxation in NT (A). Indomethacin did not inhibit bradykinin‐induced relaxation in PE (B) or pravastatin‐treated PE (E) arteries alone or in combination with LN/ODQ (B). In MβCD‐treated arteries from both NT and PE pregnancies, LN/ODQ did not significantly alter responses to bradykinin compared to MβCD alone (C, D). Data, mean ± SEM of diameter normalized to baseline (in zero [Ca^2+^]); ‘*n*’ in parentheses, each from different patients. *significant from control; *p* < 0.05, two‐way ANOVA with Šídák's post hoc multiple comparisons test. *Interaction effect relative to control. See also Table S3.

The next set of studies addressed the roles of nitric oxide (NO) and prostanoids in endothelium‐dependent vasodilation, using pharmacological inhibitors of the enzymes producing or mediating the effects of these agents. Inhibition of NO‐mediated effects (NOS, by L‐NAME) and sGC (by ODQ), or prostanoid synthesis by COX (using indomethacin), significantly reduced sensitivity to bradykinin‐induced relaxation in arteries from NT, but not PE pregnancies (Figure 3A,B; Table S4), suggesting the ability of NO and prostanoids to relax uterine arteries in PE was lost. Furthermore, inhibition of NOS and sGC alone (in the absence of vasopressin) caused constriction of arteries from NT, but not PE women (Figure S1), supporting the idea of lost NO‐mediated signaling in the latter. Combined inhibition of NOS, sGC, and COX did not further reduce sensitivity to bradykinin‐induced endothelium‐dependent relaxation in NT or PE pregnancies compared with the agents individually, suggesting convergence of these NO‐ and prostanoid‐mediated smooth muscle relaxing pathways (Figure 3A,B; Table S4). Incubation in pravastatin restored sensitivity of bradykinin‐induced relaxation to NO inhibition in arteries from PE pregnancies (Figure 3E; Table S4). Once again, block of COX alone or in combination with NOS and sGC inhibition did not further inhibit bradykinin‐endothelium‐dependent relaxation in arteries from PE pregnancies incubated with pravastatin (Figure 3E; Table S4). In contrast, in MβCD‐treated arteries from NT and PE pregnancies, NOS and sGC inhibition did not alter responses to bradykinin (Figure 3C,D; Table S4), suggesting cholesterol‐rich regions of the cell membranes are not associated with these NO and prostanoid signaling mechanisms.

**FIGURE 4:**
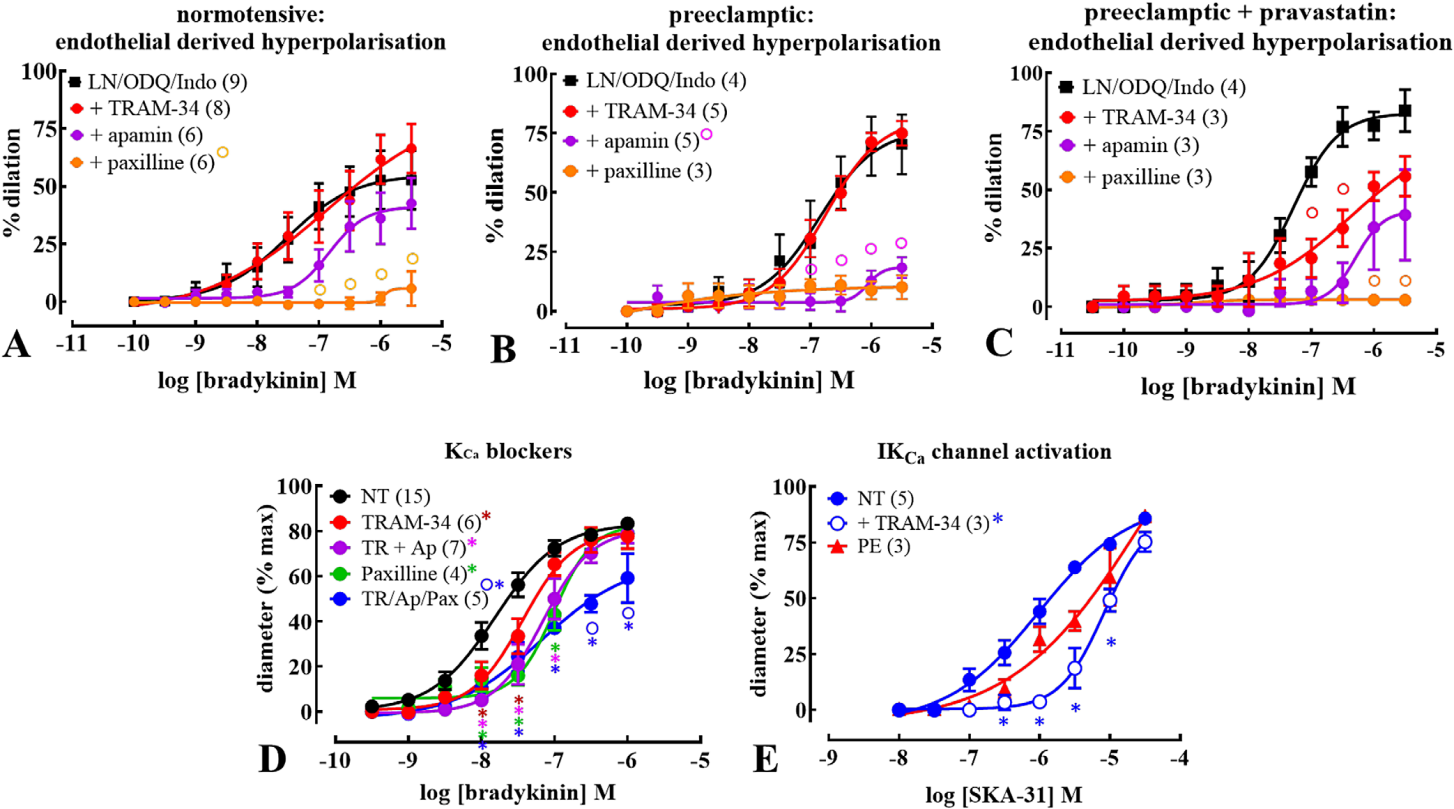
Pravastatin increases IK_Ca_‐ and BK_Ca_‐dependent relaxation in uterine radial arteries from preeclamptic (PE) pregnancies, where SK_Ca_‐mediated hyperpolarization predominates. Concentration‐ relaxation relationships for bradykinin in isolated, pressurized (60 mmHg) pre‐constricted (arginine vasopressin; 10 nM) normotensive (NT; A), PE (B), and PE + pravastatin (C) uterine radial arteries from pregnancy; and concentration‐ relaxation relationships for NT in response to SKA‐31 (D; naphtho[1,2‐*d*]thiazol‐2‐ylamine). Characterization of endothelium‐derived hyperpolarization (EDH) was performed initially in the presence of *N*_ω_‐nitro‐L‐arginine methyl ester HCl (L‐NAME; LN; 100 μM/30 min), 1H‐[1,2,4]oxadiazolo[4,3‐a]quinoxalin‐1‐one (ODQ; 10 μM/30 min), and indomethacin (Indo; 10 μM/30 min). Blockers of EDH included 1‐(2‐Chlorophenyl)diphenylmethyl‐1H‐pyrazole (TRAM‐34; TR; 1 μM/45 min), apamin (Ap; 100 nM/30 min), and paxilline (Pax; 0.3 μM/30 min) which inhibit IK_Ca_, SK_Ca_, and BK_Ca_, respectively. In NT pregnancies, IK_Ca_ inhibition by TRAM‐34 alone had no effect on untreated arteries (A). The combination of IK_Ca_ and SK_Ca_ inhibition blocked bradykinin‐induced relaxation in PE, but not NT arteries (B). In PE, SK_Ca_ block with apamin abolished the majority of bradykinin‐induced relaxation (B); not observed in NT pregnancies (A). BK_Ca_ inhibition abolished remaining relaxation in NT arteries but had no effect in PE arteries (A, B). In pravastatin‐treated PE arteries, IK_Ca_ inhibition attenuated bradykinin‐induced relaxation, with no further inhibition by SK_Ca_, while BK_Ca_ inhibition abolished the remaining relaxation (C). In NT pregnancies, IK_Ca_ or BK_Ca_ inhibition alone caused a rightward shift in bradykinin responses, and combined IK_Ca_/SK_Ca_ block showed no additional effect compared to IK_Ca_ inhibition alone. Complete IK_Ca_/SK_Ca_/BK_Ca_ block further inhibited relaxation (D). Activation of IK_Ca_ with SKA‐31 caused concentration‐dependent relaxation in NT and PE arteries, which was inhibited by TRAM‐34 in NT (E). Data, mean ± SEM of diameter normalized to baseline (in zero [Ca^2+^]), ‘*n*’ in parentheses, each from different patients. Asterisks (*) indicate significant from NT control, and circles (○) significant from the previous blocker. *Indicate significant interaction effect relative to control, and ^○^a significant interaction effect relative to previous blocker. *p* < 0.05, two‐way ANOVA with Šídák's post hoc multiple comparisons test. See also Table S4.

A significant proportion of the endothelium‐dependent dilation of arteries from both NT and PE women remained after the effects of NO and prostanoids were blocked, implying a role for EDH of vascular smooth muscle in bradykinin‐induced arterial relaxation. As EDH depends on K_Ca_ subtype activity, the role of such channels in endothelium‐dependent dilation was determined using selective channel inhibitors. In the presence of NO, sGC and COX inhibition, additional inhibition of intermediate K_Ca_ (IK_Ca_) by TRAM‐34 (IK_Ca_ inhibitor [34, 45, 46]) alone had no effect in untreated arteries from NT or PE pregnancies (Figure 4A,B; Table S4). The further inhibition of small‐(S)K_Ca_ with apamin [47] inhibited bradykinin‐endothelium‐dependent relaxation in arteries from PE, but not NT pregnancies (Figure 4A,B; Table S4). Indeed, apamin prevented the majority of the bradykinin‐induced relaxation of arteries from PE (Figure 4B; Table S4); an effect not seen in vessels from control NT patients (Figure 4A; Table S4). Subsequent large (B) K_Ca_ inhibition (by paxilline; BK_Ca_ inhibitor [48]) abolished the minor remaining bradykinin‐endothelium‐dependent relaxation in arteries from NT, but not PE pregnancies (Figure 4A,B; Table S4).

In pravastatin‐treated arteries from PE pregnancies, TRAM‐34 (in the presence of NO and prostanoid inhibition) now significantly attenuated bradykinin‐induced endothelium‐dependent relaxation, in contrast with NT and untreated PE arteries; with subsequent apamin producing no further inhibition (Figure 4C; Table S4). Paxilline abolished the remaining response in pravastatin‐treated arteries from PE patients (Figure 4C; Table S4).

To clarify K_Ca_ contribution, their individual effects in radial arteries from NT pregnancies were determined. (Figure 4D; Table S4). Inhibition of IK_Ca_ or BK_Ca_ alone caused a rightward shift of the bradykinin concentration‐response curve. Concomitant S/IK_Ca_ block did not differ to IK_Ca_ inhibition alone, with combined S/I/BK_Ca_ block increasing inhibition of bradykinin‐induced endothelium‐dependent relaxation, compared to combined S/IK_Ca_ block alone (Figure 4D; Table S4). Activation of IK_Ca_ with SKA‐31 (an IK_Ca_ agonist [49]) caused a concentration‐dependent relaxation of pre‐constricted arteries from NT and PE pregnancies, with this effect being inhibited by TRAM‐34 (Figure 4E; Table S4). Blocker effects on basal diameter were per Figure S1.

### 2.4 Expression and Localization of Endothelial Signaling Proteins in Uterine Radial Arteries

**FIGURE 5:**
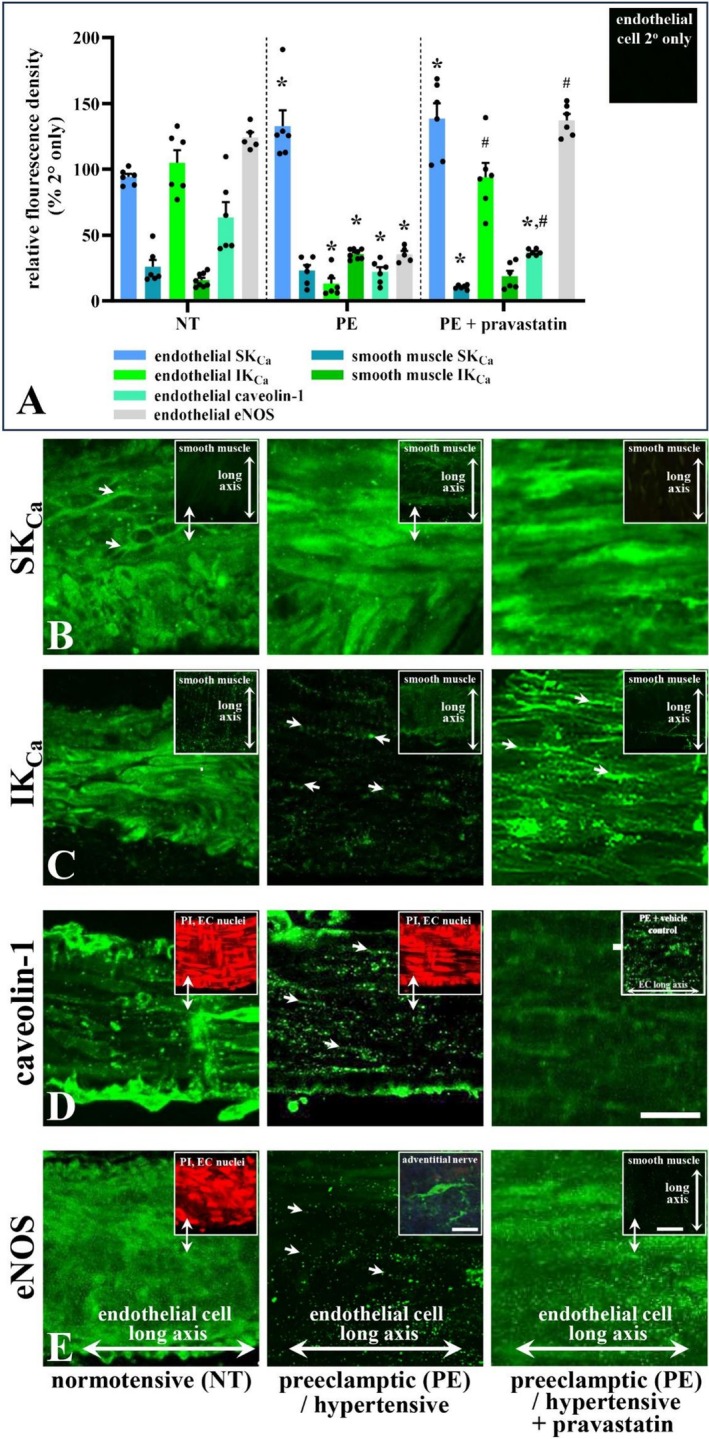
Uterine radial artery endothelial (EC) and smooth muscle cell relative fluorescence density (RFD; A) of small and intermediate conductance calcium‐activated potassium channels (S/IK_Ca_), caveolin‐1, and endothelial NOS (eNOS) immunohistochemical expression in normotensive (NT) and preeclamptic (PE) pregnancies, and pravastatin‐treated PE tissues. In NT arteries, SK_Ca_/SK3 localized to EC borders (B, arrows; *n* = 6), with EC expression increased by ~29% in PE pregnancies (C; PE *cf/*. NT; A). Diffuse smooth muscle expression was comparable between NT and PE, although low‐level puncta were more evident in PE (B, insets; A). In pravastatin‐treated PE arteries, SK_Ca_ expression was unchanged and reduced by ~56% at endothelial smooth muscle borders, respectively. Endothelial IK_Ca_/IK1/SK4 expression in NT arteries (C; *n* = 6) decreased by ~88% in PE, where punctate staining localized to endothelial borders (C, arrows; A). Smooth muscle border IK_Ca_ was present in both NT and PE arteries (C, inset; *n* = 8). Following pravastatin treatment, IK_Ca_ expression increased and decreased by ~86 and ~49% at endothelial and smooth muscle borders, respectively. Diffuse NT endothelium caveolin‐1 expression (D; *n* = 6) had RFD ~65% less so with increased puncta at endothelial borders in PE. Diffuse caveolin‐1 RFD increased following pravastatin treatment of PE tissues, but remained lower than NT. Vehicle‐treated PE controls displayed caveolin‐1 localization comparable to PE alone (D, upper inset). Diffuse eNOS in NT arteries was reduced in PE (E; A). In pravastatin‐treated PE arteries, diffuse eNOS density increased relative to both PE and NT (E; A; *n* = 6). Endothelial NOS was absent in smooth muscle (E, insets). Semi‐quantitative RFD values were normalized to secondary‐only (A, inset). Propidium iodide (PI; red) highlights nuclear localization and vessel orientation. A 10‐fold peptide excess abolished primary antibody signal (not shown). Double arrows indicate corresponding regions or focal planes. ‘*n*’, different patient tissues. Significance: *p < 0.05 vs. NT; #*p* < 0.05 vs. PE + pravastatin (A); unpaired *t*‐test, mean ± SEM. Bar, 50 μm; E, nerve,^77^ central inset (positive control), bar 10 μm.

Immunofluorescence studies were performed to assess the expression and spatial distribution of K_Ca_ subtypes to clarify if there is an association with their functional alteration in uterine radial arteries from PE compared with NT pregnancies. Expression of endothelial SK_Ca_ in arteries (Figure 5A,B) from NT patients was diffuse and associated with cell borders (Figure 5B). In contrast, in arteries from PE patients, overall SK_Ca_ density was ~29% higher and spread across the cell (Figure 5A,B), with pravastatin incubation not altering this expression (Figure 5A,B). These expression changes support the increased functional role of SK_Ca_ observed in arteries from PE women. Expression of SK_Ca_ was low‐diffuse in radial artery smooth muscle from NT pregnancies (Figure 5B, inset), being comparable to those from PE, with low‐level puncta (localized expression at a discrete‐point [18, 50]) in the latter (Figure 5B insets; Figure 5A). Post‐pravastatin incubation, PE smooth muscle cell SK_Ca_ expression was decreased ~56% at smooth muscle cell borders (Figure 5B, insets; Figure 5A).

Diffuse artery endothelial IK_Ca_ expression decreased by ~88% in arteries from PE compared with NT pregnancies (Figure 5C), with punctate endothelial cell border expression in PE (Figure 5A,C); increasing post‐pravastatin incubation by ~86% (Figure 5A,C). These expression changes also reflect the decreased functional role of IK_Ca_ observed in arteries from PE women. Expression of IK_Ca_ occurred at smooth muscle cell borders and diffusely in arteries from NT (Figure 5C, inset) and PE patients (Figure 5A,C); and decreased ~49% at smooth muscle cell borders post‐pravastatin incubation in vessels from PE (Figure 5C, insets; Figure 5A).

Further, and in a similar manner to K_Ca_, the expression of specific upstream regulators and structural proteins, including caveolin‐1 and eNOS, was determined, as NOS inhibition was ineffective on dilation in PE arteries, and caveolin‐containing, cholesterol‐rich structures in the cell membrane are relevant to the potential mechanism of pravastatin effects. Expression of endothelial caveolin‐1 is diffuse in arteries from NT pregnancies (Figure 5D), and decreased ~65% in those from PE (Figure 5A,D), with additional puncta associated with endothelial cell borders in the latter tissue. In pravastatin‐treated arteries from PE patients, diffuse endothelial‐caveolin‐1 density increased ~39% and reduced ~42% compared with PE‐ and NT‐untreated arteries, respectively (Figure 5A,D). Taken together, these observations suggest caveolin‐rich structures (by extension, caveolae) were depleted in PE arteries but restored by pravastatin.

Endothelial NOS (eNOS) expression was diffuse in arteries from NT pregnancies (Figure 5A,E), and decreased ~75% in those from PE where punctate label predominates (Figure 5A,E). In pravastatin‐treated arteries from PE patients, eNOS was diffuse and punctate, with density increased ~7 and ~76% compared with arteries from PE‐ and NT women (Figure 5A,E), respectively. Endothelial NOS was absent in smooth muscle (Figure 5E, insets). Again, these observations correlate with functional data.

**FIGURE 6:**
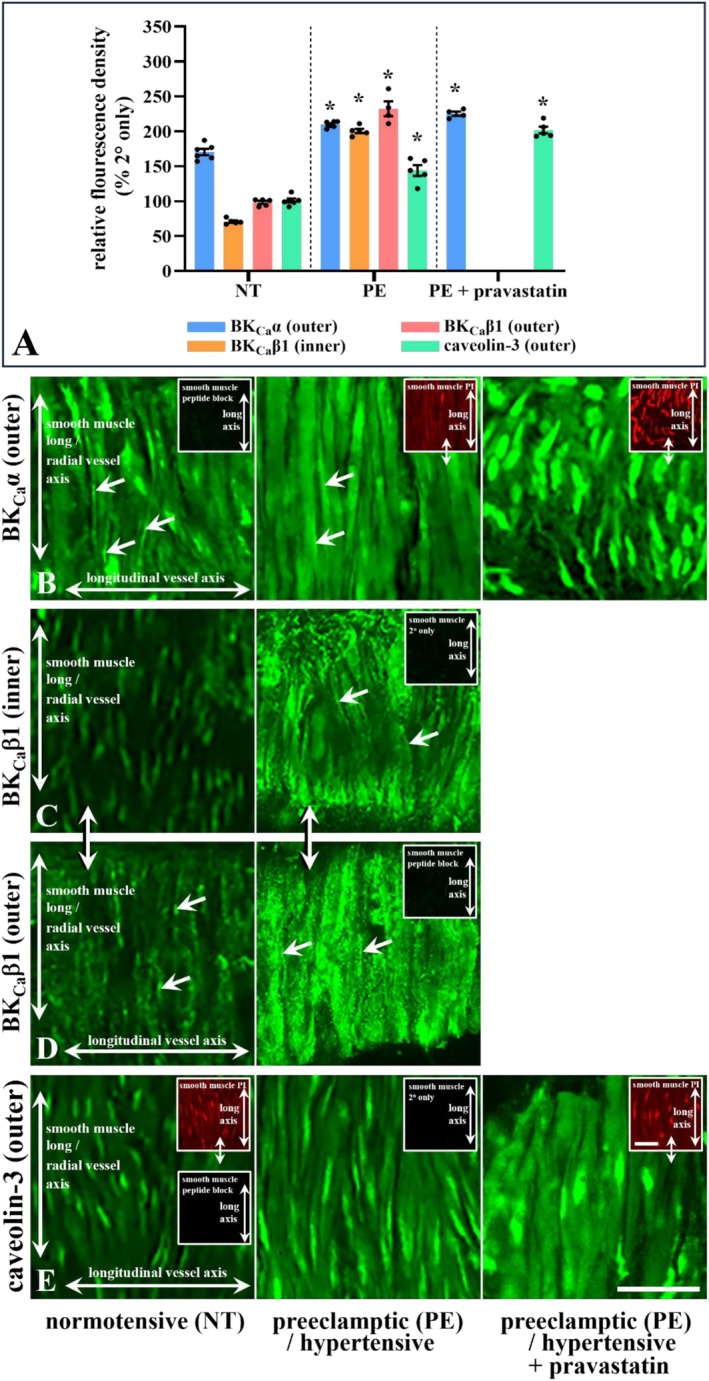
Uterine radial artery smooth muscle cell relative fluorescence density (RFD; A) of BK_Ca_α, BK_Ca_β1, and caveolin‐3 immunohistochemical expression in normotensive (NT) and preeclamptic (PE) pregnancies, and pravastatin‐treated PE tissues. Variable intensity diffuse BK_Ca_α occurred in smooth muscle cells of arteries from NT (B; *n* = 6), PE (B; *n* = 6), and pravastatin‐treated PE tissues (B; *n* = 4), predominantly at the ablumenal layer and did not differ between lumenal and ablumenal layers (data not shown). In contrast, BK_Ca_β1 exhibited layer‐specific distribution in NT arteries, with diffuse and diffuse plus punctate expression at the lumenal and ablumenal surfaces, respectively (C, D; *n* = 5). In PE arteries, diffuse and punctate BK_Ca_β1 occurred at both surfaces, with increased ablumenal puncta density (C, D; *n* = 5 and 4). Focal localization at or near smooth muscle cell borders was observed in some cells (B–D; arrows). BK_Ca_α and BK_Ca_β1 were absent from endothelial cells (data not shown). Diffuse caveolin‐3 expression occurred in smooth muscle from NT and PE arteries and in pravastatin‐treated PE tissues (D; *n* = 6, 5, and 4, respectively). Semi‐quantitative RFD values were normalized to secondary‐only (C, E insets). Propidium iodide (PI; red) highlighted nuclear localization, vessel orientation and medial layer integrity. A 10‐fold peptide excess abolished primary antibody label (B, D insets; lower inset E). Double arrows indicate corresponding vessel regions shown with alternative labelling. ‘*n*’, different patient tissues. Significance: *p < 0.05 vs. NT (A); unpaired *t*‐test, mean ± SEM. Bar, 25 μm.

Clarifying a potential structural‐functional role, BK_Ca_ distribution was determined. Diffuse and punctate BK_Ca_ expression of variable density occurred in different smooth muscle cells in radial arteries with no difference in expression at ablumenal (outer) and lumenal (inner) smooth muscle layers (Figure 6). Overall ablumenal BK_Ca_α expression density did not differ between arteries from NT and PE pregnancies, or with pravastatin‐incubated PE tissue (Figure 6A,B). Smooth muscle‐BK_Ca_β1 had different spatial distribution at inner (Figure 6C) compared to outer smooth muscle cell layers in arteries from NT patients, but not PE (compare Figure 6A,C,D); being diffuse at inner (Figure 6C), and diffuse and punctate at outer (Figure 6D) smooth muscle layers for NT. Diffuse and punctate BK_Ca_β1 occurred with greater overall expression compared with NT at both inner and outer medial layers in arteries from patients with PE (Figure 6A,C,D); with puncta density increased at the outer layer (Figure 6D). BK_Ca_α and β1 was absent in endothelial cells (*data not shown*).

### 2.5 Caveolae‐Related Protein‐mRNA Expression in Uterine Radial Arteries

Given that transcriptional changes in caveolar structural components may underlie changes in their protein, mRNA quantification of caveolae structural proteins was determined. Quantification of mRNA expression for caveolae‐related proteins, including caveolins (Cav) and cavins using rt‐qPCR showed no change in caveolin‐1 and ‐2, or cavin‐1 and ‐2 in arteries from PE compared with NT pregnancies (Figure S2). However, caveolin‐3 mRNA expression was increased in arteries from PE compared with NT pregnancies (Figure S2). Noting a potential role for caveolin‐3 in smooth muscle, its distribution and expression therein was determined. Thus, diffuse smooth muscle‐caveolin‐3 was increased in untreated and pravastatin‐treated arteries from PE compared with NT patients (Figure 6A,E).

Antibody specificity control data is per Table S5 and Supporting Information, Materials and Methods.

### 2.6 Caveolae and Myoendothelial Gap Junction Localization and Density in Uterine Radial Arteries

Endothelial caveolae density and morphology were characterized, to examine whether changes in these cholesterol‐rich membrane microdomains parallel functional changes. Untreated arteries from PE patients had lower endothelial cell caveolae density and increased caveolar flattening compared with those from untreated NT pregnancies (Figures S3–S5; Table S6). Pravastatin incubation increased lumenal membranous and submembranous caveolae density in endothelial cells of arteries from PE pregnancies, while decreasing lumenal/ablumenal and membranous and submembranous caveolae density in arteries from NT patients (Figures 3, 4, 5; Table S6). However, the density of ablumenal and membranous and submembranous caveolae in arteries from PE pregnancies was not affected by pravastatin incubation (Figures S4 and S5; Table S6).

To determine whether cholesterol depletion alone mirrored pravastatin effects, the impact of MβCD treatment was assessed. Incubation in MβCD had similar caveolae depleting effects as pravastatin in arteries from NT pregnancies (Figure S5). In MβCD and pravastatin‐treated arteries from NT and PE pregnancies, endothelial caveolae flattening/disruption increased above untreated control (Figures S3 and S4). In MβCD‐treated arteries from PE pregnancies, lumenal submembranous caveolae density increased by ~50% compared with untreated PE arteries, while ablumenal membranous and submembranous caveolae density increased relative to both untreated and pravastatin‐treated PE arteries (Figures S3–S5; Table S6).

Noting the dependence of EDH on heterocellular electrical coupling, myoendothelial gap junction density was determined. The density of myoendothelial gap junctions decreased ~64% in arteries from PE compared with NT patients (Figure S6; Table 2); with pravastatin‐treatment of arteries from PE patients increasing myoendothelial gap junction density in these vessels to a level similar to NT (Table 2).

**TABLE 2:** Uterine radial artery myoendothelial gap junction density.

	Normotensive (NT; *4*)	Preeclampsia (PE; *4*)	PE + pravastatin (*3*)
	4.68; 4.48; 7.95; 7.33	0.67; 1.24; 3.58; 3.32	5.17; 4.62; 5.33
Mean ± SEM	**6.1 ± 0.9**	**2.2 ± 0.7** *	**5.0 ± 0.2**

* Significant from NT control; number of myoendothelial gap junctions per 5 μm vessel length; unpaired *t*‐test *p* < 0.05; mean ± SEM. *n*, parentheses, each from different patients.

## 3 Discussion

In isolated uterine radial arteries from PE compared to NT pregnancies, endothelium‐dependent relaxation was impaired, predominantly due to the loss of NO and BK_Ca_‐mediated signaling, with concomitant decreased myoendothelial gap junction and caveolae density. Incubation of arteries from PE pregnancies in pravastatin restored bradykinin‐induced relaxation to NT levels through increased contribution of NO, IK_Ca_, and BK_Ca_, with a related *increased* endothelial caveolae and myoendothelial gap junction density, and the latter returning to levels in arteries from NT pregnancies. Thus, the potential beneficial effects of pravastatin on radial artery blood flow appear related to distinct changes in mechanisms of endothelium‐dependent relaxation, rather than direct changes in membrane cholesterol content.

In the ex vivo uterine radial arteries from NT pregnancies, endothelial SK_Ca_ plus prostanoids and NO underlie endothelium‐dependent smooth muscle relaxation [2, 20, 51, 52]. The present study shows an additional role for BK_Ca_ in endothelium‐dependent relaxation of these arteries from NT pregnancies. In smooth muscle of radial arteries from NT and PE pregnancies, differential expression of BK_Ca_α and β_1_ subunits occurred, consistent with previous reports of channel upregulation in pregnancy, although notably this pertained to ovine fourth‐order uterine arteries [53] and mouse primary uterine arteries [54]. Albeit, the functional implications of this are debated, with altered BK_Ca_α‐β_1_ interaction being a potential dysfunctional mechanism for BK_Ca_ activity (per discussion [55]). In radial arteries from PE patients, dysfunction of endothelium‐dependent relaxation involves loss of NOS/COX‐ and BK_Ca_‐mediated activity, despite significant smooth muscle BK_Ca_α and ‐β1 expression, which increased relative to NT, with comparable responses to the NO‐donor SNP in arteries from NT pregnancies.

In human small uterine arteries (~150–250 μm), presumably similar to those of the present study (as they are the same caliber), from NT and PE pregnancies, Luksha et al. suggested a switch in endothelium‐dependent relaxation activity from cytochrome P450 enzyme‐ to hydrogen peroxide‐mediated that may influence smooth muscle BK_Ca_ function in PE, compared to NT [20]. In light of the present data, which demonstrates restorative effects of pravastatin on smooth muscle BK_Ca_ function, a potential mechanism of action may also involve reactivation of cytochrome P450 enzyme‐dependent pathways. Alternatively, or in addition, altered BK_Ca_‐mediated relaxation is consistent with changes in hydrogen sulfide‐dilator action in pregnancy, where downregulation of its primary enzyme (cystathionine‐γ‐lyase) in PE has been suggested [56, 57]. A notable factor contributing to differences in endothelium‐mediated dilator mechanisms is elevated BMI predominant in PE (per table 1, and table 1 in each of [5, 20]), where previous animal studies have shown, for example, a switch in endothelium‐dependent dilation from primarily NO to myoendothelial gap junction and IK_Ca_‐derived EDH [58]. Further work is required to clarify this issue in human pregnancy.

Notably, as a component of endothelium‐dependent relaxation, increased NO activity has been suggested in human small uterine radial arteries in PE [5]. However, the present findings are consistent with the suggestion of a reduced NO component in endothelium‐dependent relaxation in uterine arteries from PE pregnancies [20]. Vasodilation responses to the NO donor sodium nitroprusside were similar in all arteries and treatment groups, suggesting changes in NO‐mediated dilation were caused by altered eNOS activity and/or NO bioavailability. This potentially indicates a role for reactive oxygen species, peroxynitrite, and inflammatory cytokines. Notably, clinical data on vascular NO levels in PE vary, with suggested increase [59, 60]; and reduced bioavailability proposed, with the latter attributed to decreased synthesis and degradation of reactive oxygen species [61, 62].

In the present study, decreased myoendothelial gap junction density in uterine radial arteries from PE *cf*. NT pregnancies may result in limiting the local spread of EDH to smooth muscle thereby reducing endothelium‐dependent relaxation, consistent with suggestions in similar sized (~250 μm diameter) uterine arteries in PE [20, 63, 64]. This is further consistent with the present data where pravastatin incubation increased myoendothelial gap junction density and associated contribution of localized IK_Ca_ to endothelium‐dependent relaxation in arteries from PE pregnancies to levels compared to arteries from NT pregnancies, demonstrating plasticity in heterocellular coupling, as a mechanism present in many vascular beds in health, that can be modified in disease [18, 25].

In arteries from PE pregnancies, endothelial SK_Ca_ expression is increased and redistributed and accompanies SK_Ca_‐dominant (bradykinin‐induced) endothelium‐dependent relaxation. Endothelial‐SK_Ca_ is at a low level on the membrane and at higher levels at cell borders in arteries from animal models [50, 65], similar to their elevation in radial arteries from PE patients in the present study, and consistent with their colocalization with endothelial cell border gap junctions [50, 65]. Typically, these junctions occur within ≤ ~1 μm of myoendothelial gap junctions [63], with this proximity likely facilitating efficient EDH transfer (to smooth muscle), and conduction of membrane potential and its related change in diameter over distance [66]. Dysfunction of coordinated homo‐ and heterocellular gap junction signaling occurs in essential hypertension [63, 67]; and provides a potential link to PE and the correction of deficient endothelium‐dependent relaxation and excess blood pressure by pravastatin. Relative myoendothelial gap junction density has been shown to be proportional to EDH and NO activity; with increased or decreased density being associated with the contribution of these factors to vessel relaxation [68]. Pravastatin increased IK_Ca_‐EDH‐mediated relaxation of smooth muscle in arteries from PE pregnancies, with it thus acting at interrelated pathways in a pleiotropic manner (see also ref [16]). Here, this includes enhancing gap junction‐dependent endothelium‐dependent relaxation; noting that absence of specific gap junction inhibitors precludes their functional examination here [69].

Decreased eNOS in arteries from PE compared to NT is consistent with lesser NO contribution in the disease state, as is elevated NO activity and eNOS expression in vessels from PE women treated with pravastatin. Endothelial NOS is negatively regulated by caveolin‐1, and thus its reduced expression/downregulation in PE is a potential mechanism for increasing eNOS activity, per human *placental* vessels [70, 71]. However, this effect was absent in uterine radial arteries in the present study, where vessels from PE patients displayed reduced caveolin‐1 expression and caveolae density comparable to those from NT pregnancies, which paradoxically paralleled loss of NO endothelium‐dependent relaxation; presumably as eNOS activity was impaired. Present functional data suggest that pravastatin treatment restores eNOS function in uterine radial arteries from PE pregnancies, which is speculated to occur through decreased reactive oxygen species production and increased eNOS coupling. Anatomical evidence of pravastatin‐induced caveolae upregulation in arteries from PE pregnancies suggests increased eNOS post‐pravastatin necessitates the restoration of caveolae to modulate NO availability, with further work required to clarify this. Of note, there are many intrinsic differences in uterine and placental arteries, and at birth, the latter organ is pathophysiological; and thereby not reflective of healthy activity.

Cholesterol depletion via MβCD, which directly extracts cholesterol from the plasma membrane, produced similar caveolae flattening in both NT and PE tissues to that of pravastatin, which acts intracellularly via the HMG‐CoA reductase pathway, albeit produced converse effects on caveolae density in arteries from NT and PE pregnancies. These latter data suggest that cholesterol metabolism and its interaction with structural caveolae proteins differ in arteries from NT and PE pregnancies, and likely underlie the differing functional responses to cholesterol‐targeting agents, as MβCD caused no functional improvement in arteries from PE pregnancies, despite increased caveolae density and flattening, suggesting cholesterol removal alone is insufficient and pleiotropic effects of pravastatin are key. Indeed, the present study showed MβCD induced caveolae remodeling within a ≤ 1 h incubation window, suggesting a time course for functional and expression changes with this organelle. Given that expression of caveolin and cavin mRNA was unchanged in arteries from PE and NT, except for upregulation of Cav‐3 in smooth muscle, caveolae and caveolin‐1 related changes in PE likely occur via post‐translational modification (noting Table S7, primer properties).

### 3.1 Therapeutic implications

This study shows that ex vivo uterine radial artery endothelium‐dependent relaxation occurs via overlapping, complex, but distinct pathways in arteries from NT *cf*. PE pregnancies. The work identifies potential mechanisms of acute pravastatin‐mediated action in correcting endothelium‐dependent relaxation in uterine radial arteries in PE pregnancies. The use of pravastatin in ameliorating vascular uterine dysfunction in PE supports its potential therapeutic application to improve vascular function in patients with PE, reducing hypertension and improving organ perfusion; although its role(s) in PE prevention requires further investigation.

### 3.2 Limitations

Examining and determining the cellular and biochemical signaling mechanisms in uterine radial arteries from patients that have been taking pravastatin in a clinical trials setting (e.g., NCT01717586) as the in situ state, reflects a key future direction of the present work; particularly in the context of confirming the validity of the present in vitro/ex vivo data, and how it may improve endothelial dysfunction in PE. Concurrently, a key potential limitation of the present work is the in vitro/ex vivo incubation of human tissue in pravastatin, and how well it may reflect the in situ state.

Additional limitations of the present study primarily relate to further work. In the same cohorts, albeit in tissue from women taking pravastatin clinically, it would be ideal to determine the:

- Nature of the homo‐ and heterocellular (myoendothelial) gap junction coupling and coordination of uterine vascular signaling; particularly noting their association with endothelial S/IK_Ca_ in resistance arteries from animal models [18].
- Calcium signaling pathways associated with EDH‐type and NO signaling herein.
- Mechanisms of smooth muscle and endothelium‐dependent constriction.
- Changes in endothelial membrane potential as a key mechanism underlying vascular tone and blood flow. As the stimulus‐mediated reduction in endothelial membrane potential, EDH results in vasodilatory changes in artery diameter (pedantically referred to) as EDH‐type activity (e.g., Refs. [18, 72]).
- Potential developmental effects on function and expression data, due to the ~16% gestational age difference (Table 1) of NT *cf/PE‐derived* radial arteries.
- Potential effects of limited sample sizes, of *n* = 14 and 10 for NT and PE, respectively (Table 1, inclusive of 1 female twin PE birth).
- Potential for effects of variation in radial artery collection site between patients, and with specific surgeons.
- Functional and expression data in radial arteries from NT pregnancies incubated with pravastatin; noting preliminary anatomical data indicate no change in S/I/BK_Ca_ or caveolin‐1 expression in such vessels (Sandow, Luque and Murphy, unpublished results).
- Potential influence of fetal sex on PE pathology and offspring health [45, 73]. While data herein were analyzed with regard to fetal sex, numbers were too small to clarify this point.

## 4 Materials and Methods

Detailed methods are available as Supporting Information.

## 5 Conclusions

Preeclampsia involves uterine microvascular endothelial dysfunction, and although its mechanism of action is unknown, in early trials, pravastatin treatment improves vascular function in the disease [9, 14]. This study is the first to examine the effects of pravastatin on endothelium‐dependent relaxation of human uterine arteries from women with PE. The work shows that acute pravastatin treatment restores (and potentially overcompensates) for the impaired endothelium‐dependent dilation in isolated uterine radial arteries from PE patients to levels seen in vessels from NT pregnancies. Pravastatin enhances NO synthase activity, increases I/BK_Ca_ function, and restores myoendothelial gap junction density, whereby uterine vasodilator dysfunction is alleviated primarily via these non‐cholesterol‐lowering effects of pravastatin (see also ref [16]). Thus, the present study clarifies the potential mechanisms that underlie endothelial dysfunction in PE and how they may be targeted and corrected in the maternal uterine circulation. The ability of pravastatin to reverse PE‐induced loss of NO‐ and I/BK_Ca_‐mediated endothelial signaling in PE supports the potential use of this statin in the preventative treatment of PE as a disease driven, at least in part, by vascular endothelial dysfunction. The present work advances understanding of vasodilator signaling mechanisms that control blood flow and pressure in the uterus and blood supply to the placenta and fetus, and their modification and potential correction in disease.

## Author Contributions

**Nathan M. Luque:** conceptualization, data curation, formal analysis, investigation, methodology, project administration, validation, visualization, writing – original draft preparation, writing – review and editing. **Leo Leader:** conceptualization, data curation, methodology, project administration, resources, validation, writing – review and editing. **Sandra M. Lowe:** methodology, resources, writing – review and editing. **Steven D. Horrowitz:** methodology, resources, writing – review and editing. **Marianne Tare:** methodology, resources, writing – review and editing. **Victoria Hinkley:** investigation, methodology, resources, validation, writing – review and editing. **Vladimir V. Matchkov:** methodology, writing – review and editing. **Maged M. Costantine:** conceptualization, methodology, writing – review and editing. **Irit Markus:** methodology, writing – review and editing. **Lu Liu:** methodology, writing – review and editing. **Shaun L. Sandow:** conceptualization, data curation, formal analysis, investigation, methodology, project administration, resources, supervision, validation, visualization, writing – original draft preparation/review and editing. **Timothy V. Murphy:** conceptualization, data curation, formal analysis, investigation, methodology, project administration, resources, supervision, validation, visualization, writing – original draft preparation/review and editing.

## Funding

This work was supported by the University of the Sunshine Coast, SPARK fund to S.L.S.

## Ethics Statement

Ethical approval for the study was from the South‐Eastern Sydney Local Health District and University of New South Wales Human Research Ethics Committees (approvals 14/219; LNR/14/POWH/495), REGIS 2019/ETH04978, conforming to the principles outlined in the Declaration of Helsinki.

## Consent

Human experiments were approved by institutional review boards and participants gave informed consent.

## Conflicts of Interest

The authors declare no conflicts of interest.

## Supporting information


**Figure S1:** Basal diameter in the presence of enzyme and channel blockers.
**Figure S2:** qPCR analysis of caveolae‐related protein mRNA.
**Figure S3:** Endothelial cell caveolae properties in uterine radial arteries from normotensive and preeclamptic (PE) pregnancies.
**Figure S4:** Endothelial cell caveolae flattening is increased in uterine radial arteries of preeclamptic (PE) patients *cf*/ normotensive, and is increased in both following methyl‐β‐cyclodextrin or pravastatin incubation.
**Figure S5:** Endothelial cell caveolae density in uterine radial arteries from normotensive and preeclamptic (PE) pregnancies.
**Figure S6:** Myoendothelial gap junctions and associations in uterine radial arteries from normotensive pregnancies.
**Figure S7:** Proposed mechanisms of eNOS uncoupling in preeclampsia.
**Table S1:** Myometrial radial artery constriction with arginine vasopressin.
**Table S2:** Myometrial radial artery dilation with sodium nitroprusside.
**Table S3:** Myometrial radial artery dilation with bradykinin.
**Table S4:**. Myometrial radial artery dilation associated with EDH channel activation/contribution.
**Table S7:**, which outlines the rt‐qPCR primer (sequence) design that were generated by Sigma‐Aldrich (St Louis).
**Table S5:** Antibody characteristics.
**Table S6:**. Uterine radial artery endothelial caveolae density per μm vessel length.
**Table S7:** rt‐qPCR primers.

## Data Availability

Data are available from the corresponding author upon reasonable request.
