# Supplementary material for: Pravastatin Corrects Endothelial Dysfunction in Ex Vivo Uterine Radial Arteries in Preeclampsia
Source: Acta Physiol (Oxf). 2026 Mar 11;242(4):e70186. doi: 10.1111/apha.70186 (PMC12976587; doi:10.1111/apha.70186)
Supplement: Supplementary file 1 — Figure S1: Basal diameter in the presence of enzyme and channel blockers. Figure S2: qPCR analysis of caveolae‐related protein mRNA. Figure S3: Endothelial cell caveolae properties in uterine radial arteries from normotensive and preeclamptic (PE) pregnancies. Figure S4: Endothelial cell caveolae flattening is increased in uterine radial arteries of preeclamptic (PE) patients cf/ normotensive, and is increased in both following methyl‐β‐cyclodextrin or pravastatin incubation. Figure S5: Endothelial cell caveolae density in uterine radial arteries from normotensive and preeclamptic (PE) pregnancies. Figure S6: Myoendothelial gap junctions and associations in uterine radial arteries from normotensive pregnancies. Figure S7: Proposed mechanisms of eNOS uncoupling in preeclampsia. Table S1: Myometrial radial artery constriction with arginine vasopressin. Table S2: Myometrial radial artery dilation with sodium nitroprusside. Table S3: Myometrial radial artery dilation with bradykinin. Table S4:. Myometrial radial artery dilation associated with EDH channel activation/contribution. Table S7:, which outlines the rt‐qPCR primer (sequence) design that were generated by Sigma‐Aldrich (St Louis). Table S5: Antibody characteristics. Table S6:. Uterine radial artery endothelial caveolae density per μm vessel length. Table S7: rt‐qPCR primers. [file APHA-242-e70186-s001.docx]

**Supporting Information**

**Materials and Methods**

Vessel function and morphology, and related protein/mRNA expression were characterised using pressure myography (**Table S1-S4**), confocal immunohistochemistry (antibodies, **Table S5**), electron microscopy (EM; **Table S6**), and qPCR (primers; **Table S7**), corresponding to **Figure S1** (function), qPCR (**Figure S2;** anatomy), ultrastructure (**Figures S3-S7**; anatomy), per the **Tables** and **Figures** in the main manuscript.

**Participant recruitment, and sample collection and preparation.** Patients were prospectively recruited at the Royal Hospital for Women based on clinical diagnosis at the time of admission for delivery or gynaecological surgery. Diagnostic criteria for preeclampsia were consistent with Royal Australian and New Zealand College of Obstetrics and Gynaecology definitions at the time of recruitment (i.e. new-onset hypertension ≥140/90 mmHg after 20 weeks’ gestation with proteinuria ≥300 mg/day or other maternal organ dysfunction). Ethical approval for the study was obtained from the South Eastern Sydney Local Health District and University of New South Wales Human Research Ethics Committees (approvals 14/219; LNR/14/POWH/495), REGIS 2019/ETH04978, conforming to the principles outlined in the Declaration of Helsinki. Written informed consent was obtained pre-operatively following provision and discussion of a participant information sheet, with the opportunity to withdraw at any time. Hence, samples were obtained from consenting NT and PE pregnant patients undergoing caesarean-section deliveries, with patient medications noted.

The anatomical and functional characteristics of endothelium-dependent dilatory control of *ex vivo* uterine radial arteries in NT and PE pregnant patients, and the effects of acute pravastatin (pravastatin sodium salt hydrate; Sigma, Cat# P4498) therein were determined. On the basis that pravastatin is a cholesterol-depleting agent, the effect of another such agent, Methyl-β-cyclodextrin (MβCD; Sigma, Cat# M7439), on arteries was also examined as a baseline for assessing the direct effects of cholesterol removal on vessel anatomy and function. Uterine radial arteries from NT and PE patients were pre-incubated with pravastatin (2 mM/6h;[^1^](#_ENREF_1)^,^[^2^](#_ENREF_2)), MβCD (10 mM/1h) *in vitro,* or vehicle. The specific incubation concentration and time-frames used reflects the consolidation of information from existing studies.[^2-5^](#_ENREF_2)

Avoiding any obvious venous sinuses, a sample of myometrium was taken from the superior aspect of the lower uterine segment incision once the fetus had been safely delivered, and there was no excessive bleeding. Inner uterine wall segments, ~10 x 10 mm were placed in a sterile container of dissection buffer (4°C; mM:3 MOPS, 145 NaCl, 5 KCl, 2.5 CaCl_2_, 1 MgSO_4_, 1 NaH_2_PO_4_, 0.02 EDTA, 2 pyruvate, 5, 1% BSA). The surgery was done by a number of different surgeons, with all instructed in the procedure by one of the authors. With hypertension (blood pressure >140/90 mmHg), PE was defined by the inclusion of significant proteinuria, or end organ dysfunction, with or without proteinuria, occurring >20 wks gestation.[^6-8^](#_ENREF_6)

Third order uterine radial arteries (identified as being ~150-250 µm internal diameter) were dissected from the myometrium; for mRNA work were snap-frozen in liquid nitrogen, stored at -80°C; separately fixed for immunohistochemistry and EM, per standard procedures,[^9-12^](#_ENREF_9) and stored at 4°C. Functional studies were performed on freshly isolated arteries within 24 hrs of collection. Notably, some individual preparations were used for single, and some for multiple methodologies (i.e. different segments of the same artery were cut into separate segments).

Pregnant patient exclusion criteria were essential hypertension, diabetes, history of smoking and biliary disease.[^6-8^](#_ENREF_6) Control NT samples were without essential hypertension, diabetes, history of smoking or biliary disease. Notably, no patient had diabetes, established atherosclerosis, chronic hypertension, malignancy, hepatic or renal failure, systemic infection, autoimmune diseases, and recent surgery or trauma.

**Pressure myography.** Artery segments were placed in modified Krebs solution in a 6ml pressure myograph chamber, cannulated at both ends on glass micropipettes and secured with 10-0 nylon suture, per previous.[^13^](#_ENREF_13)^,^[^14^](#_ENREF_14) Cannulated arteries were coupled to a pressure-servo controller with peristaltic pump (Living Systems, PS-200, USA), superfused with modified Krebs solution (3 ml/min, 34°C) and checked for leaks by assessing pressure at 120 mmHg for 30 s. Vessels were equilibrated at 40 mmHg before raising pressure to 60 mmHg, with studies performed in the absence of intra-luminal flow.[^15^](#_ENREF_15)^,^[^16^](#_ENREF_16)

A concentration of 3 or 10 nM **arginine vasopressin** (arg^8^-vasopressin; activator of vasopressin 1 receptors on vascular smooth muscle[^15^](#_ENREF_15); Sigma, Cat# V9879) pre-constricted vessels to ~50% of the initial artery diameter. In some arteries, a full concentration-response curve to arginine vasopressin (0.1 nM-0.1 µM) was first conducted. In pre-constricted arteries, concentration-dilation relationships were obtained to **bradykinin** (bradykinin acetate salt; 0.1 nM-10 µM; endothelium-dependent vasodilator acting on B_2_ receptors[^17-19^](#_ENREF_17); Sigma, Cat# B3259), **sodium nitroprusside** (sodium nitroferricyanide(III) dihydrate; NO donor that directly activates soluble guanylate cyclase in smooth muscle[^17-19^](#_ENREF_17); Sigma, Cat# 228710) or **SKA-31** (0.1-30 µM; naphtho[1,2-d]thiazol-2-ylamine; an S/IK_Ca_ agonist[^20^](#_ENREF_20); Sigma, Cat#S5576), followed by several bradykinin concentration-dilation curves with the sequential addition of enzyme- and channel inhibitors (≤5 concentration-dilation relationships per segment); **L-NAME** (Nω-nitro-L-arginine methyl ester HCl; 100 µM/30 min; endothelial NO synthase (NOS) blocker [^18^](#_ENREF_18)^,^[^19^](#_ENREF_19)^,^[^21^](#_ENREF_21); Sigma, Cat# 5751), **ODQ** (1H-[1,2,4]oxadiazolo[4,3-a]quinoxalin-1-one; 10 µM/30 min; soluble guanylate cyclase (sGC) inhibitor[^18^](#_ENREF_18)^,^[^19^](#_ENREF_19)^,^[^21^](#_ENREF_21); Sigma, Cat# O3636), **indomethacin** (cyclooxygenase (COX) inhibitor; 10 µM/30 min, blocking prostacyclin synthesis[^22^](#_ENREF_22); Sigma, Cat# I7378); **TRAM-34** (1-(2-chlorophenyl)diphenyl)methyl]-1H-pyrazole; 1 µM/45 min; IK_Ca_ inhibitor [^15^](#_ENREF_15)^,^[^16^](#_ENREF_16)^,^[^23^](#_ENREF_23); Toronto Research Chemicals, Cat# T705100), **apamin** (0.1 µM/30 min; SK_Ca_ inhibitor [^24^](#_ENREF_24); Sigma, Cat#A9459); **paxilline** (0.3 µM/30 min; BK_Ca_ inhibitor[^25^](#_ENREF_25); Toronto Research Chemicals, Cat# P207600). After each concentration-dilation relationship was obtained, drugs were washed out and vessels allowed to recover before being reconstricted with arginine vasopressin. At the conclusion of each experiment, arteries were incubated in nominally Ca^2+^-free Krebs solution (no added Ca^2+^ plus 2 mM/L EGTA) for 20min to determine the maximum artery diameter at 60 mmHg. Notably, phenylephrine was initially examined as a potential preconstricting agent, but per other functional studies of human resistance arteries from our lab (such as mesenterics[^13^](#_ENREF_13)), no response to this agent precluded its use.

In experiments examining the effects of acute pravastatin, vessels were pre-incubated in 2 mM/L pravastatin-Krebs solution, as above, prior to being mounted on the pressure myograph and the above experimental protocol. In MβCD experiments, mounted/pressurised arteries were confirmed for arg^8^-vasopressin response as detailed above before being intralumenally perfused through replacement of intralumenal Krebs with MβCD-Krebs solution (10 mM/1h), as above.

**Confocal immunohistochemistry.** Uterine radial artery segments were incubated in modified Krebs solution (mM: 111 NaCl, 25.7 NaHCO_3_, 4.9 KCl, 2.5 CaCl_2_, 1.2 MgSO_4_, 1.2 KH_2_PO_4_, 5.75 glucose and 10 HEPES[^11^](#_ENREF_11)^,^[^26^](#_ENREF_26)) or Krebs with pravastatin,[^27^](#_ENREF_27) as above,. and fixed in 3% paraformaldehyde in phosphate buffered saline (Sigma, P4417), pH 7.4; incubation concentration and time-frames reflects the consolidation of information from previous studies.[^2^](#_ENREF_2)^,^[^5^](#_ENREF_5) Segments were opened along one side of their long axis and pinned to Sylgard, endothelial-side ‘up’, before incubation in blocking buffer (phosphate buffered saline with 1% bovine serum albumin, 0.1% Triton X) at ~23^o^C for 2 h. Tissues were then rinsed in phosphate buffered saline, 3x5min and incubated in primary antibody (see **Table S5**) in blocking buffer, 4°C, 18 h; with further 3x5 min phosphate buffered saline rinse at ~23^o^C, followed by incubation in secondary antibody at ~23^o^C, 2 h (see **Table S5**), diluted in phosphate buffered saline containing 0.01% Triton X. A final 3 x 5 min phosphate buffered saline rinse preceded mounting tissue in anti-fade glycerol; with some containing 0.002% propidium iodide to verify cell layer patency. Examples of methods and analysis, including antibody controls, are as previous, and use cell and tissue of confirmed positive and negative expression,[^11^](#_ENREF_11)^,^[^12^](#_ENREF_12)^,^[^28^](#_ENREF_28) with autofluorescence at 488 nm visualising internal elastic lamina. Images were collected using a Nikon Eclipse Ti (RRID:SCR_021242) confocal microscope with using uniform settings, and relative fluorescence intensity determined (see also **Statistics**, below) using Photoshop (RRID:SCR_014199) and/or ImageJ/FIJI (RRID:SCR_003070). Tissues were processed in a deidentified/blinded manner, defined post-analysis. Notably relative fluorescence detection does not differentiate between diffuse and punctate signal, (see e.g.[^9^](#_ENREF_9); for review[^29-33^](#_ENREF_29)).

**Real-time qPCR -** *Primer selection and design.* mRNA expression for *CAV1, CAV2, CAV3, CAVIN1* and *CAVIN2* genes were assayed in arteries from NT and PE patients, with *GAPDH* additionally used as a housekeeping sequence. Primers were generated by Sigma-Aldrich (St Louis; sequences per **Table S7**).

Total RNA was extracted from 20 artery samples (8 NT and 12 PE from individual patients) using TRIzol reagent and homogenised with a Precellys homogeniser before chloroform addition to separate nucleic acids from vascular proteins and lipids with centrifugation. The aqueous upper phase was removed, and isopropanol added (1:1) to promote RNA pellet precipitation, which was subsequently collected and washed with 70% ethanol before resuspension in nuclease-free H_2_O. RNA yield and purity were assessed using a Nanodrop-1000 spectrophotometer. Reverse transcription extracted total RNA (2 μg) to produce single strand complementary DNA (sscDNA). PCR amplification used a Master Cycler Realplex2 (Eppendorf, Germany), with primer efficiency assessed via inclusion of a melting phase step at each run end. DNA gel electrophoresis was conducted to verify PCR product sizes. Relative gene expression (fold-change) was determined as 2^-ΔΔCt^.

**Transmission electron microscopy** **(EM).** Conventional and serial section transmission EM determined caveolae and myoendothelial gap junction density in segments of uterine radial arteries, as above, according to standard procedures.[^9^](#_ENREF_9)^,^[^11^](#_ENREF_11)^,^[^26^](#_ENREF_26) NT and PE patient samples were incubated in modified Krebs (control), with MβCD or pravastatin, as above, prior to EM processing and imaging;[^14^](#_ENREF_14) incubation concentration and time-frames reflects the consolidation of information from existing studies.[^2-5^](#_ENREF_2) Caveolae and myoendothelial gap-junction density were per previous; each ‘*n*’ as mean of 4 different randomly selected cell profiles per vessel.[^14^](#_ENREF_14) Sections were imaged in a JEOL 1100 transmission EM at 16 MP.

**Immunoelectron microscopy****.** Optimal ultrastructural and antigenic preservation used high pressure freezing, automated freeze substitution and low temperature embedding of fresh uterine radial arteries from NT patients in Krebs buffer; per standard procedures.[^9^](#_ENREF_9) In brief, unfixed fresh tissue segments were frozen at high pressure (∼2100 bar; Leica EM High Pressure Freezer; RRID:SCR_021367), freeze-substituted at -90°C (Leica Automated Freeze Substitution) in 0.2% uranyl acetate in acetone for 4 d. Samples were infiltrated and embedded in LR White (ProSciTech) at -25°C, and polymerized under ultraviolet in the freeze substitution unit (RRID:SCR_020230) at -25^o^C. Sections were then mounted on formvar (0.5% in chloroform) and carbon-coated (~5-10 nm) slot grids, incubated in blocking buffer (as above, for *immunohistochemistry*) for 30 min, followed by primary antibody (**Table S5**) in blocking buffer for 2 h at 22^o^C, followed by 5 nm Au-conjugated secondary antibody (**Table S5**) in 0.01% Tween 20 for 2 h at ~23^o^C. Tissues was processed for immunofluorescence as above, with colloidal gold conjugated secondary antibody (ProSciTech, QLD, Australia). EM imaging was as above, with controls confirmed per confocal immunohistochemistry, as above.

**Statistics.** For functional studies, diameter measurements were calculated as a percentage of maximal diameter (determined in zero Ca^2+^ Krebs at 60 mmHg) or diameter normalised to baseline and expressed as mean ± SEM; maximal diameter being determined in Ca^2+-^free Krebs, EGTA containing Krebs. Non-linear regression analysis in GraphPad Prism (Software, USA; RRID:SCR_002798) determined pEC_50_ values and maximum diameter (mean ± SEM). ‘*n*’ indicates the number of arteries from different patients. Two-way ANOVA with Sidak’s *post hoc* comparison was used to evaluate the significance of treatments; significance of pEC_50_ and maximum dilation significance was assessed via one-way ANOVA.

For each individual patient artery examined with immunohistochemistry, fluorescence density was determined in 4-6 individual areas of each vessel. Each of these 4-6 were averaged for each patient artery and the mean of these averaged as the ‘*n*’ for that antigen. Data are mean ± SEM, via unpaired *t-test.*

For qPCR of *CAV-1, CAV-2, CAV-3, CAVIN-1* and *CAVIN-*2 mRNA expression in uterine radial arteries from NT (*n* = 8) or PE (*n* = 12) patients were assessed for fold-change; with statistical significance determined with the Mann-Whitney unpaired t-test. Values of *P*<0.05 were taken as significant.

For EM analysis, 4 areas from each vessel segment for each ‘*n*’ were averaged and taken as an ‘*n*’. Tissue types and treatments were averaged for NT and PE, for weighted mean ± combined SEM across n=4 per treatment, accounting for between-subject variability; individual SEMs not shown, and two-way ANOVA with Tukey’s *post-hoc* multiple comparisons test to evaluate caveolae, and unpaired *t-test* to evaluate myoendothelial gap-junction density.

**Materials.** Except where stated, all reagents for functional experiments were from Sigma-Aldrich (St Louis, MI) and Toronto Research Chemicals (North York, CA); Confocal microscopy / EM reagents were from ProScitech (Qld, Australia).

**Supplemental Figures**

**Figure S1. Basal diameter in the presence of enzyme and channel blockers** in pressurized (60 mmHg) uterine radial arteries from normotensive and preeclamptic pregnancies (‘*n’* in parentheses). *N*_ω_-nitro-L-arginine methyl ester HCl (L-NAME; LN; 100 µM/30 min), 1H-[1,2,4]oxadiazolo[4,3-a]quinoxalin-1-one (ODQ; 10 µM/30 min), indomethacin (Indo; 10 µM/30 min). 1-(2-chlorophenyl)diphenyl)methyl]-1H-pyrazole (TRAM-34; TR; 1 µM/45 min), apamin (Ap; 100 nM/30 min), and paxilline (Pax; 0.3 µM/30 min). Columns represent mean ± SEM of diameter normalized to maximum (in zero [Ca^2+^] EGTA). *, significant difference from control; *P*<0.05, one-way ANOVA.

**
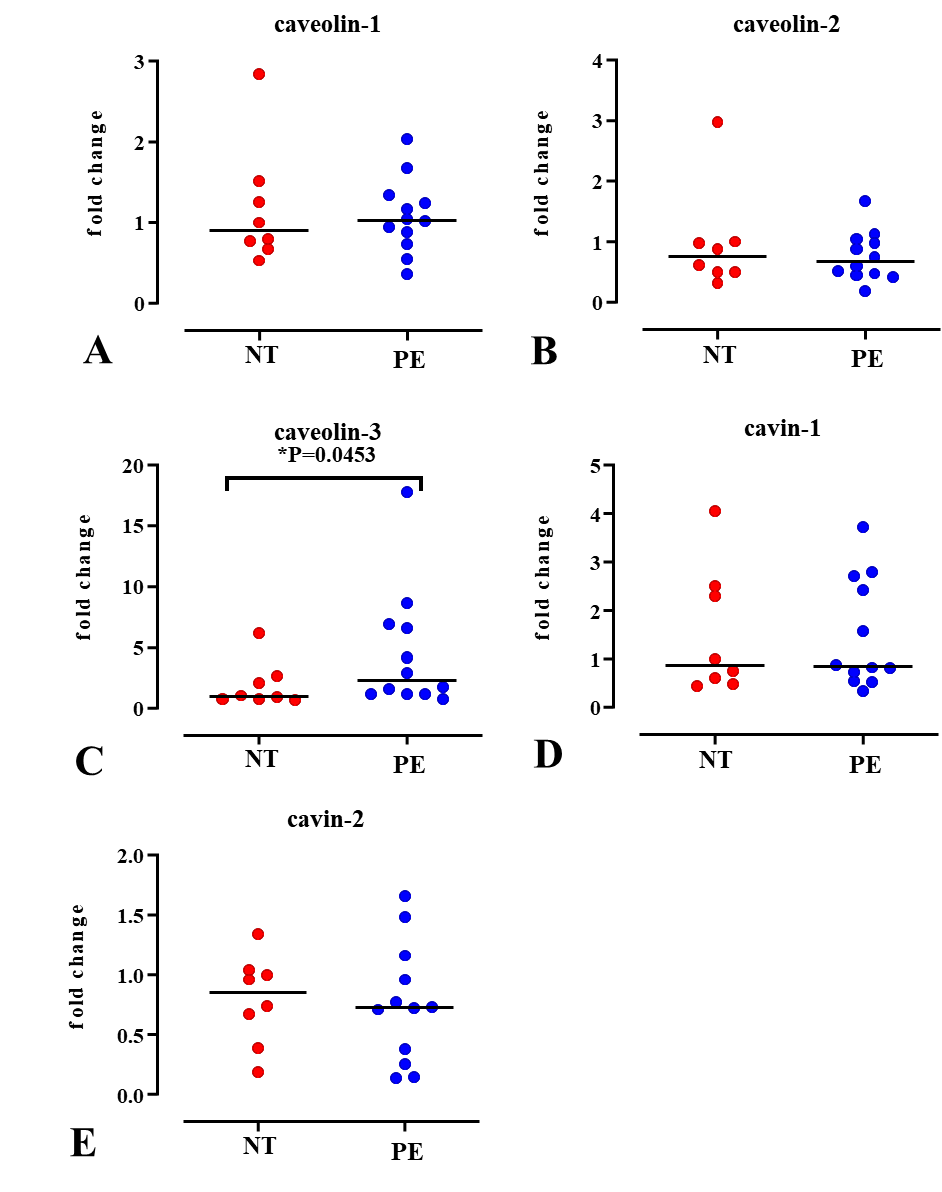
**

**Figure S2. qPCR analysis of caveolae-related protein mRNA**. The mRNA expression of caveolin-1, caveolin-2, caveolin-3, cavin-1 and cavin-2 (**A,B,C,D,E,** respectively) in uterine radial arteries from normotensive (NT) pregnant patients (*n*=8) or patients with preeclampsia (PE; *n*=12). Data are presented as median scatter plots. Caveolin-3 mRNA expression was significantly increased in tissues from PE compared to NT. *P*<0.05, Mann-Whitney unpaired t-test.

**
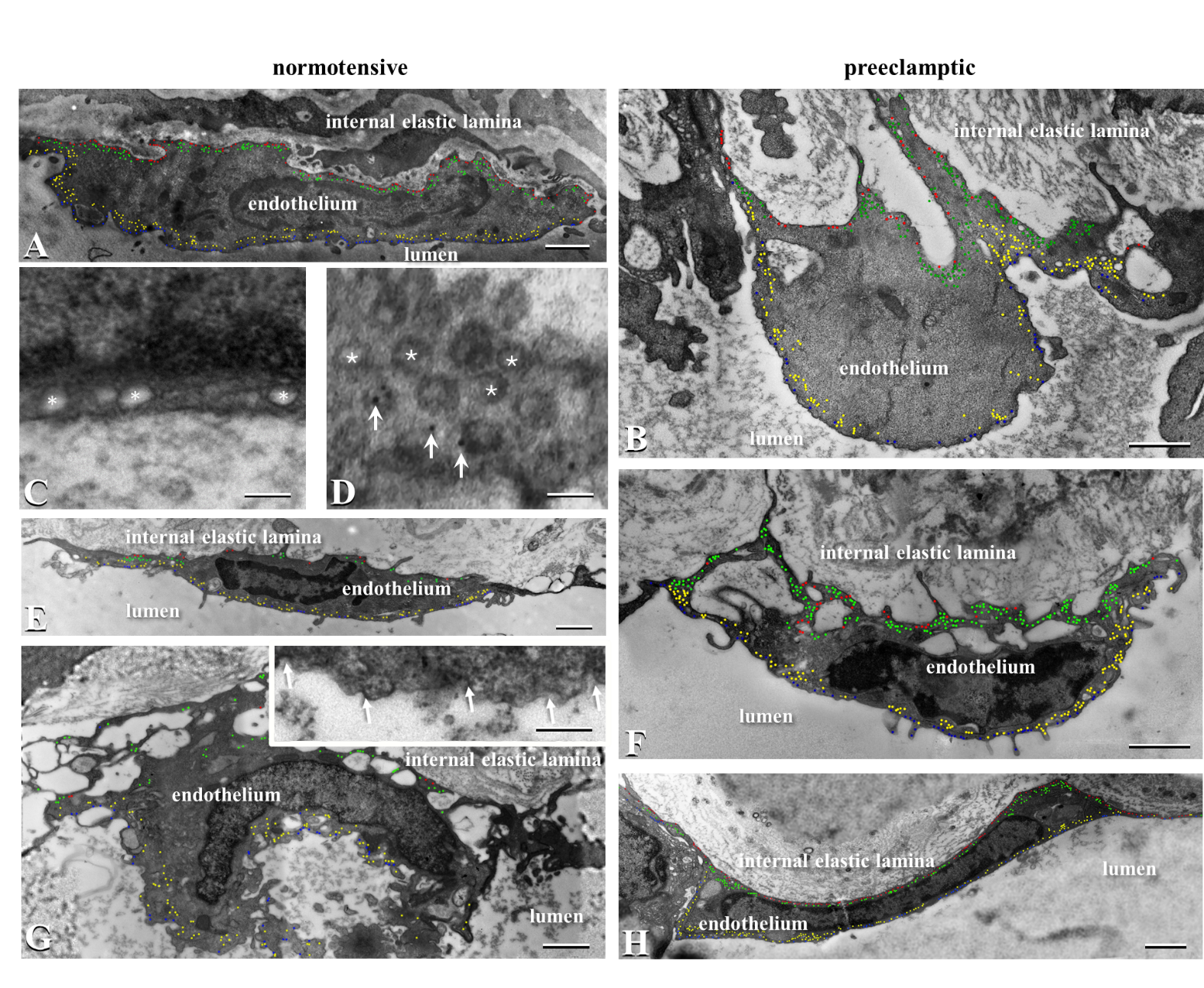
**

**Figure S3. Endothelial cell caveolae properties in uterine radial arteries from normotensive and preeclamptic (PE) pregnancies.** Caveolae density in endothelial cells in uterine radial arteries from untreated normotensive (NT; **A**), PE (**B**) pregnancies. Examples (*) of caveolae are shown (**C**); and caveolin-1-5 nm Au (secondary; arrows) illustrates caveolin-1 localization to caveolae (examples, *) in tissue from an NT patient (**D**). Caveolae shown from pravastatin (2 mM/6h) treated-NT (**E**) and -PE (**F**), as well as methyl-β-cyclodextrin (MβCD; 10 mM/1h) treated-NT (**G**) and -PE (**H**) patients; with MβCD and pravastatin associated caveolar flattening (**G**, inset). Relative to untreated controls ~17 and 8% of membranous caveolae were ‘flattened’ post-MβCD-treatment and ~10 and 10% of membranous caveolae were ‘flattened’ post-pravastatin-treatment in endothelial cell of arteries from NT and PE pregnancies, respectively (*n*=4, each for arteries from NT and PE patients). Example caveolae sites are labelled as membranous lumenal (blue), ablumenal (red); and submembranous lumenal and ablumenal caveolae (yellow and green, respectively; **A,B,E,F,G,H**). Bar, 1 µm (**A-B,E-H**), 100 nm (**C-D**), 250 nm (**G**, inset).


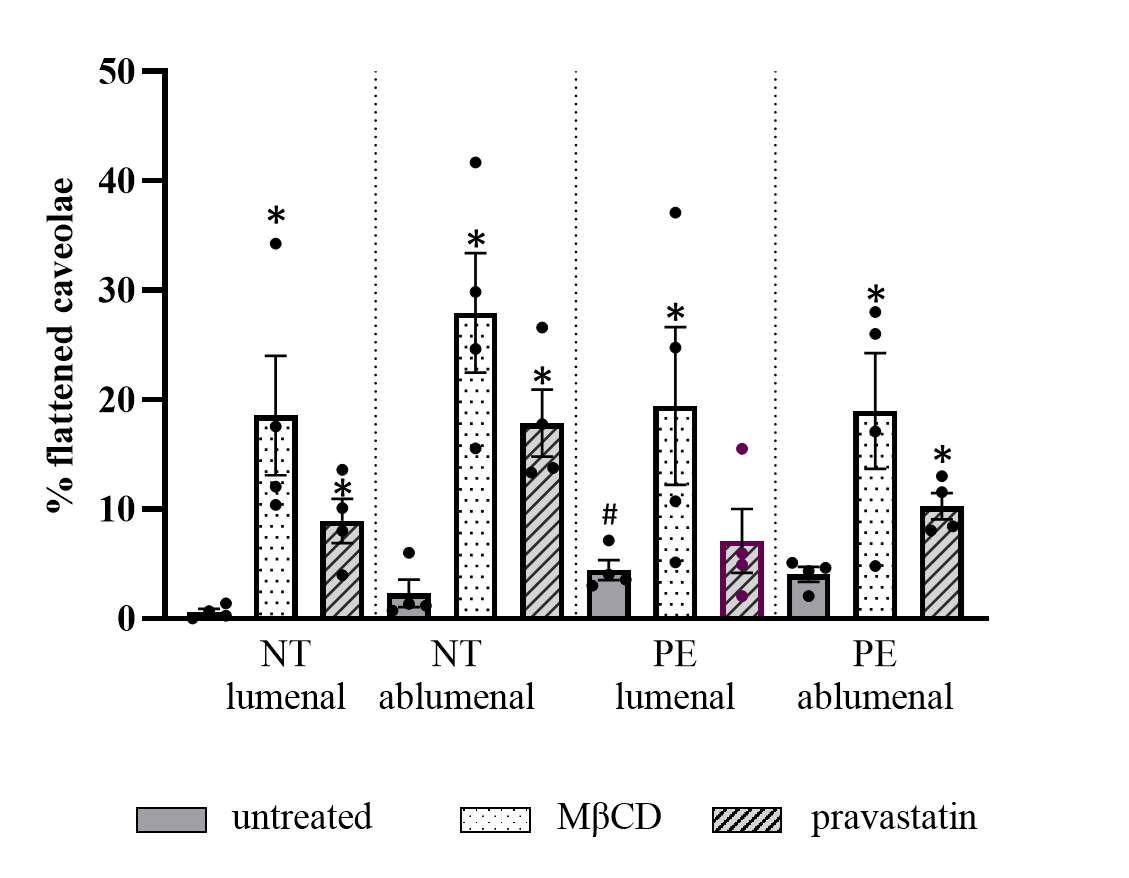


**Figure S4. Endothelial cell caveolae flattening is increased in uterine radial arteries of preeclamptic (PE) patients *cf/* normotensive, and is increased in both following methyl-β-cyclodextrin or pravastatin incubation.** The percentage of membranous endothelial cell caveolae flattening seen in PE pregnancies was increased *cf/* normotensive (NT), and the caveolar flattening effects of both methyl-β-cyclodextrin (MβCD) and pravastatin illustrated in both lumenal, and ablumenal surfaces from both cohorts, save for at the PE endothelial cell luminal surface with pravastatin. Data, mean ± SEM of *n* = 4, per treatment. *, significant difference from respective untreated tissues; #, PE untreated vs NT untreated vessels; *P*<0.05; unpaired t-test. See also **Figure S7.**


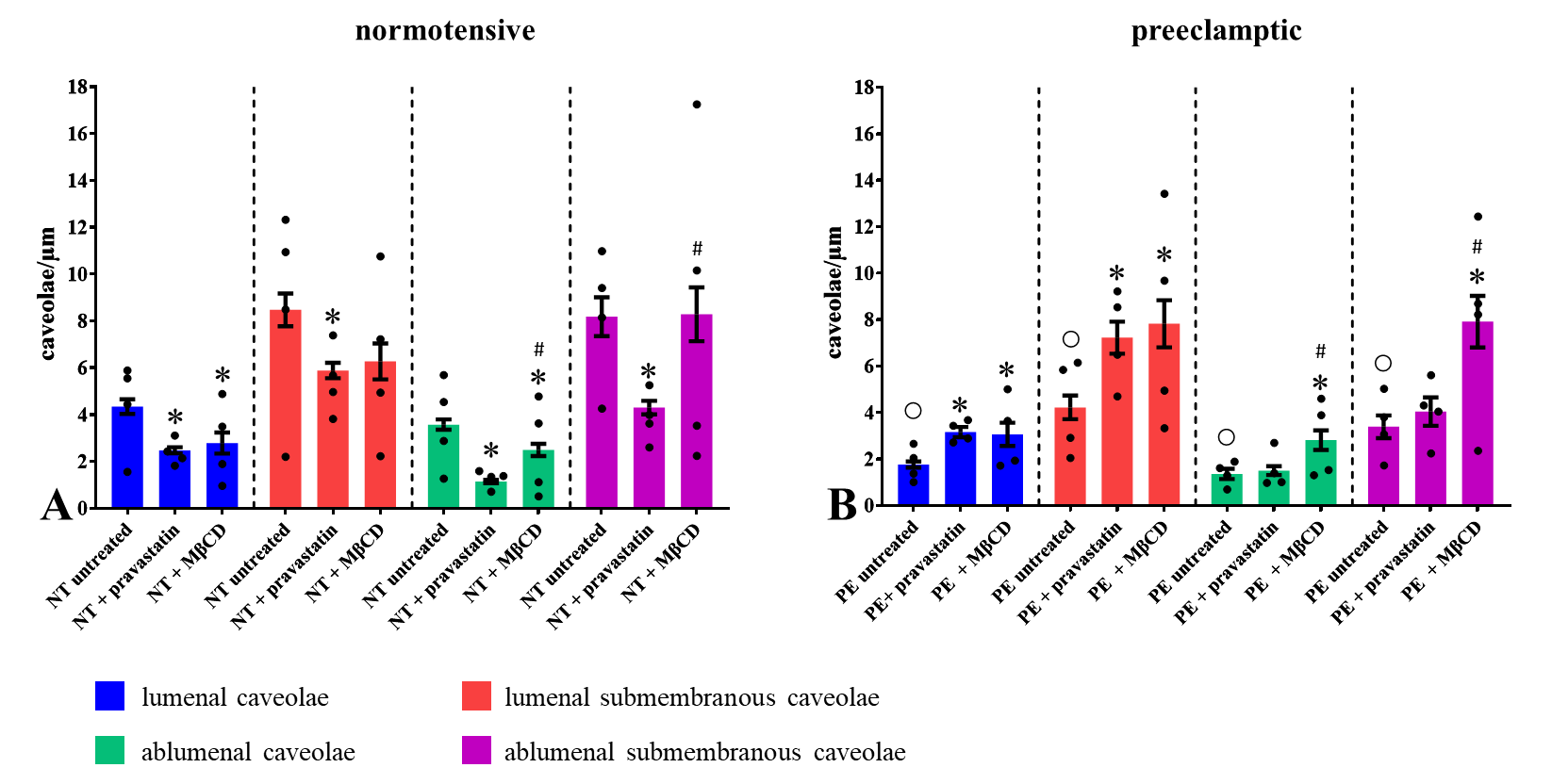


**Figure S5. Endothelial cell caveolae density in uterine radial arteries from normotensive and preeclamptic (PE) pregnancies.** Endothelial cell caveolae density (total wall length examined/μm) is higher across lumenal/ablumenal and membranous/submembranous regions from normotensive (NT; **A**) *cf/.* untreated PE pregnancies (**B**). Membranous and submembranous caveolae density is decreased across lumenal/ablumenal and membranous/submembranous regions of endothelial cells following pravastatin incubation *cf/.* untreated NT, with methyl-β-cyclodextrin (MβCD) affecting only the membranous lumenal, and membranous and submembranous ablumenal regions (**A**). Membranous and submembranous caveolae density is increased at the lumenal side of endothelial cells in PE pregnancies following pravastatin incubation, whereas increased caveolae density is seen in submembranous lumenal caveolae, and membranous and submembranous ablumenal caveolae following methyl-β-cyclodextrin (MβCD) incubation (**B**). Data are presented as weighted mean ± combined SEM across n=4 per treatment, accounting for between-subject variability; individual SEMs not shown. Asterisks (*) indicate a significance of pravastatin- or MβCD-treated PE or NT arteries *cf/.* untreated controls; circles (○) indicate a significance of untreated PE arteries *cf/.* untreated NT arteries; hashes (#) indicate significance of MβCD-treated PE or NT arteries *cf/.* pravastatin-treated counterparts; *P*<0.05; two-way ANOVA with Tukey’s *post-hoc* multiple comparisons test.

**
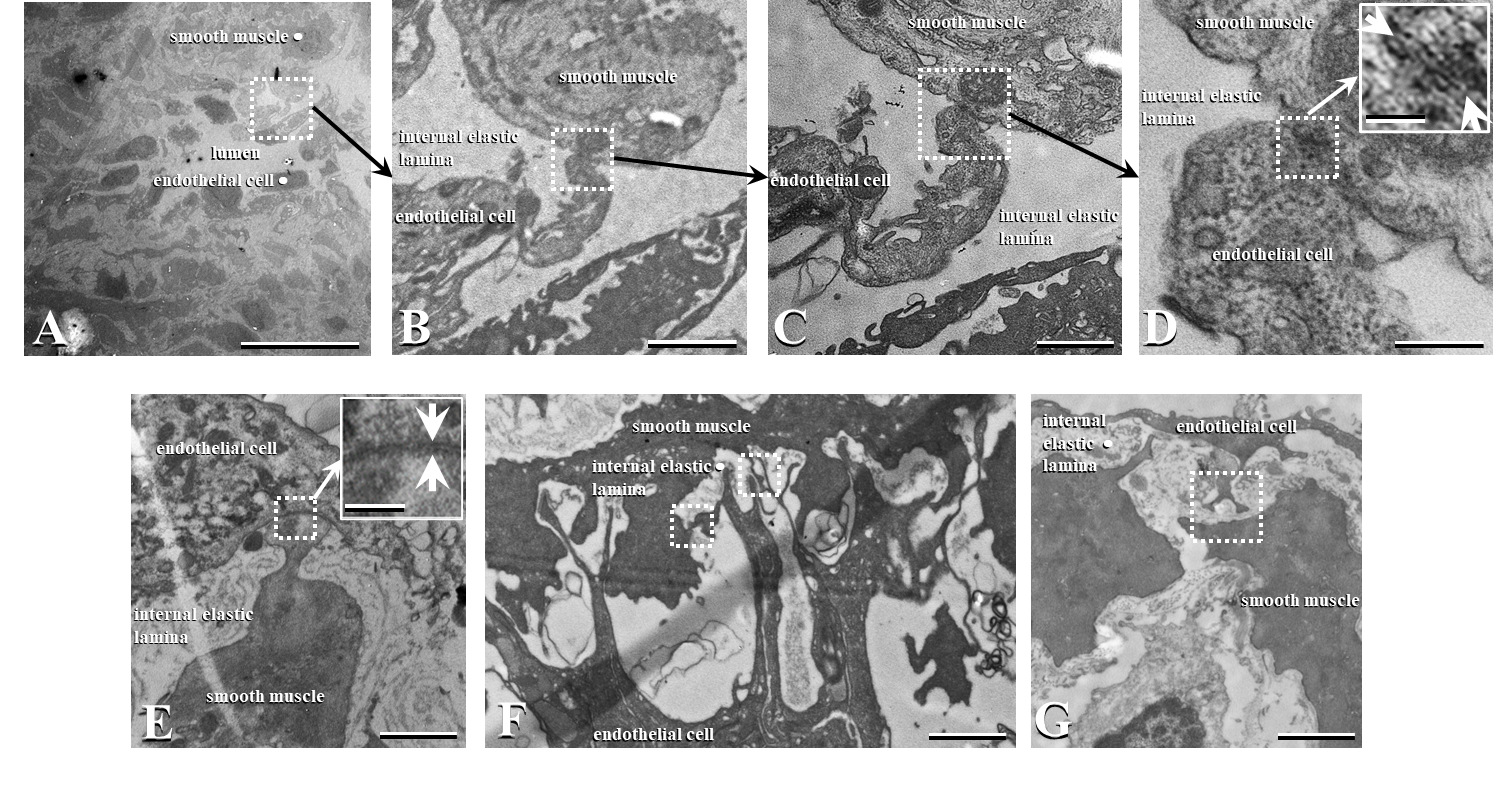
 Figure S6. Myoendothelial gap junctions and associations in uterine radial arteries from normotensive pregnancies.** Myoendothelial gap junction/contact site (dashed square; as a single vessel site at increasing magnification, **A-D**), as an endothelial cell-derived extension through the internal elastic lamina to a smooth muscle cell; **A-D**); a smooth muscle cell extension through the internal elastic lamina to the adjacent endothelial cell (**E;** e.g. dashed square and inset), and a meeting of both endothelial cell and smooth muscle cell projections (**F;** e.g. dashed squares), or myoendothelial projections through the internal elastic lamina to the adjacent smooth muscle cell (**G;** e.g. dashed square). Myoendothelial gap junctions occur as ~10 nm thick pentalaminar regions (e.g. insets, between arrows; **D,E**) where adjacent cells are separated by a ~3 nm gap in the membrane. Bar, 10 µm (**A**), 2 µm (**B**), 1 µm (**C,E-G**), 0.25 µm (**D;E**, inset), 0.1 µm (**D**, inset). Images representative of *n* = 4, each from a different patient.

**
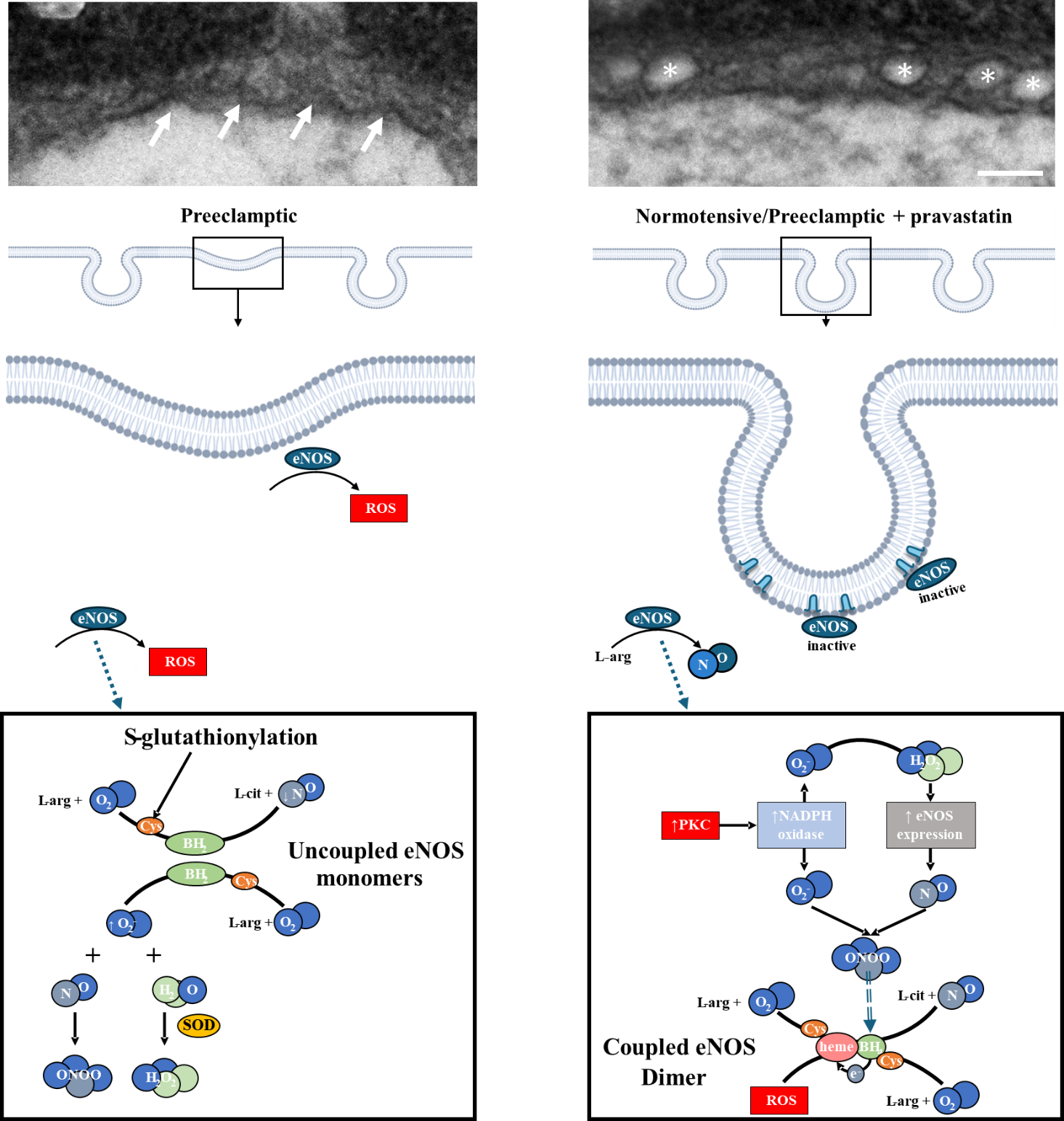
**

**Figure S7. Proposed mechanisms of eNOS uncoupling in preeclampsia.** Under normotensive conditions (right), the eNOS pathway initiates when tetrahydrobiopterin (BH4) binds to dimeric endothelial nitric oxide synthase (eNOS), promoting electron transfer to the heme group. This allows heme to bind O₂ and L-arginine (L-arg), resulting in the synthesis of nitric oxide (NO), L-citrulline (L-cit), and small amounts of reactive oxygen species (ROS). The uncoupling of eNOS and the subsequent increase in ROS production are key contributors to endothelial dysfunction. Disruption of caveolae, specialized membrane microdomains, has been shown to promote eNOS uncoupling due to their role in eNOS compartmentalization and regulation. Under normal conditions, eNOS is inactive when bound to caveolin-1, a structural protein in caveolae, which regulates NO bioavailability. In preeclampsia (PE) myometrial radial artery endothelial cells (EC), there is a simultaneous reduction in caveolae density and increased flattening of both the lumenal and ablumenal surfaces compared to normotensive (NT) controls. This could be an adaptive response to increase NO bioavailability; however, if unchecked, it may predispose to eNOS uncoupling and endothelial dysfunction. Uncoupling occurs under conditions of increased NADPH oxidase activity, resulting in the formation of peroxynitrite (ONOO¬-). Additionally, eNOS uncoupling in PE may occur through S-glutathionylation of NOS cysteine residues (Cys), facilitated by non-enzymatic, glutathione S-transferase, or NO-promoted pathways. Peroxynitrite oxidizes BH_4_ to a trihydrobiopterin radical (BH_3_), which dismutates into 6,7-[8H]-H2-biopterin (BH_2_). In the absence of BH4, the bond between O₂ and heme is disrupted, leading to eNOS-mediated superoxide (O₂⁻) production. Superoxide can react with NO to form ONOO⁻ or undergo enzymatic conversion by superoxide dismutase (SOD) to produce hydrogen peroxide (H₂O₂). This increases ROS production, further driving BH_4_ oxidation and eNOS uncoupling, amplifying oxidative stress. The reduced caveolae density and increased flattening of caveolae in PE suggest a greater proportion of activated eNOS. While this may enhance NO bioavailability, the ROS-rich environment in PE predisposes to excessive ROS production. These molecular and structural disruptions collectively contribute to endothelial dysfunction in PE. In PE radial artery, endothelial caveolae (e.g. asterisks) flatten (e.g. arrows) compared to those in NT. Caveolae density increases in PE + pravastatin (upper image panels) with flattening only increased on the ablumenal surface. Bar, 100 nm. See also **Figures S3-5.**

**Supplemental Tables**

**Table S1.** **Myometrial radial artery constriction with arginine vasopressin.**

|  | ‘*n*’ | pEC50 ± SEM | % constriction at 10nM normalised to max ± SEM | % max constriction ± SEM |
| --- | --- | --- | --- | --- |
| Normotensive | *11* | 8.7 ± 0.3 | 59.0 ± 5.3 | 53.6 ± 5.5 |
| Normotensive + MβCD | *5* | 9.4 ± 0.7 | 71.9 ± 5.3 | 67.2 ± 7.9 |
| Preeclamptic | *5* | 9.0 ± 0.4 | 45.0 ± 7.2 | 39.7 ± 7.0 |
| Preeclamptic + MβCD | *3* | 8.7 ± 0.1 | 67.1 ± 10.7 | 57.6 ± 6.7 |
| Preeclamptic + pravastatin | *4* | 8.8 ± 0.1 | 51.1 ± 6.9 | 54.4 ± 10.6 |
| Interaction effect assessed via two-way ANOVA with Šídák's *post hoc* multiple comparisons test; pEC50, constriction at 10 nM and max constriction significance assessed via one-way ANOVA with Šídák's *post hoc* multiple comparisons test; *P*<0.05 taken as for all statistical tests. MβCD, methyl-β-cyclodextrin. ‘*n*’ each from a different patient. See also Figure 1. | | | | |

**Table S2.** **Myometrial radial artery dilation with sodium nitroprusside.**

|  | ‘*n*’ | pEC50 ± SEM | % max normalised to baseline ± SEM |
| --- | --- | --- | --- |
| Normotensive | *8* | 7.2 ± 0.2 | 77.9 ± 5.2 |
| Preeclamptic | *7* | 7.2 ± 0.3 | 80.1 ± 5.9 |
| Preeclamptic + pravastatin | *5* | 7.0 ± 0.6 | 91.9 ± 3.8 |
| Normotensive + MβCD | *5* | (I) 9.20 ± 0.5  (II) 7.2 ± 0.1 | 67.8 ± 16.7 |
| Preeclamptic + MβCD | *5* | (I) 9.6 ± 0.4 (II) 6.4 ± 0.3 | 80.5 ± 12.2 |
| Assessed via two-way ANOVA with Šídák's *post hoc* multiple comparisons test; pEC50 and max dilation significance assessed via one-way ANOVA with Šídák's *post hoc* multiple comparisons test; *P*<0.05 taken as for all statistical tests. I and II are the pEC50 for each half of the biphasic response for those respective curves. MβCD, methyl-β-cyclodextrin. ‘*n*’ each from a different patient. See also Figure 2. | | | |

**Table S3.** **Myometrial radial artery dilation with bradykinin.**

|  | blocker(s) | ‘*n*’ | pEC50 ± SEM | % max normalised to baseline ± SEM |
| --- | --- | --- | --- | --- |
| Normotensive | control | *15* | 7.8 ± 0.1 | 83.3 ± 2.1^●^ |
|  | indomethacin* | *9* | 7.2 ± 0.2* | 76.9 ± 8.9 |
|  | L-NAME/ODQ* | *7* | 7.4 ± 0.1* | 67.1 ± 7.1 |
|  | + indomethacin* | *9* | 7.5 ± 0.3 | 52.8 ± 12.7* |
|  | + TRAM-34* | *8* | 6.9 ± 0.9 | 66.4 ± 10.7 |
|  | + apamin* | *6* | 6.8 ± 0.2* | 42.7 ± 11.0* |
|  | + paxilline*^○^ | *6* | 5.9 ± 31.9 | 5.7 ± 7.5*^○^ |
| Normotensive + MβCD | MβCD* | *9* | 8.5 ± 0.4 | 79.7 ± 9.9 |
|  | + L-NAME/ODQ* | *6* | 9.0 ± 0.8 | 55.9 ± 12.4 |
| Preeclamptic (PE) | control* | *10* | 8.1 ± 0.2 | 59.6 ± 8.6* |
|  | indomethacin | *6* | 7.4 ± 0.1 | 69.7 ± 15.9 |
|  | L-NAME/ODQ | *7* | 7.7 ± 0.2 | 71.6 ± 8.1 |
|  | + indomethacin | *4* | 6.9 ± 0.3 | 70.3 ± 12.6 |
|  | + TRAM-34 | *5* | 6.7 ± 0.1^●^ | 74.9 ± 5.2 |
|  | + apamin^●○^ | *5* | 6.1 ± 0.2^●^ | 18.3 ± 4.2^●○^ |
|  | + paxilline | *3* | NC | 10.9 ± 4.0^●^ |
| Preeclamptic + MβCD | MβCD | *4* | 7.4 ± 0.6 | 77.0 ± 23.0 |
|  | + L-NAME/ODQ | *4* | 7.0 ± 0.7 | 60.5 ± 13.9 |
| Preeclamptic + pravastatin | control | *5* | 8.2 ± 0.2 | 93.7 ± 3.4^●^ |
|  | L-NAME/ODQ* | *4* | 7.3 ± 0.1 | 89.0 ± 6.2 |
|  | + indomethacin* | *4* | 7.3 ± 0.1 | 83.9 ± 9.0 |
|  | + TRAM-34* | *3* | 6.4 ± 0.9* | 55.8 ± 8.5*^○^ |
|  | + apamin* | *3* | 6.3 ± 0.3* | 40.3 ± 18.4* |
|  | + paxilline* | *3* | NC | 2.9 ± 1.8*^○^ |

*, significant from normotensive control; ●, significant from preeclamptic control; ○, significant from previous treatment. Interaction effect assessed via two-way ANOVA with Šídák's *post hoc* multiple comparisons test; pEC50 and max dilation significance assessed via one-way ANOVA with Šídák's *post hoc* multiple comparisons test; *P*<0.05 taken as for all statistical tests. NC, not calculable. ‘*n*’ each from a different patient. See also **Figure 3**.

**Table S4**. **Myometrial radial artery dilation associated with EDH channel activation / contribution.**

| Tissue | activator | blocker(s) | ‘*n*’ | pEC50 ± SEM | % max dilation ± SEM |
| --- | --- | --- | --- | --- | --- |
| Normotensive | SKA-31 | control | *5* | 6.01 ± 0.13 | 85.8 ± 2.3 |
|  |  | TRAM-34* | *3* | 5.08 ± 0.12 | 75.3 ± 4.4* |
|  | bradykinin | control | *8* | 7.76 ± 0.05 | 87.0 ± 1.0 |
|  |  | paxilline* | *4* | 7.01 ± 0.08* | 78.9 ± 2.9 |
|  |  | TRAM-34* | *6* | 7.44 ± 0.09 | 77.7 ± 5.5 |
|  |  | + apamin* | *7* | 7.16 ± 0.09* | 78.8 ± 4.2 |
|  |  | + paxilline*^○^ | *5* | 7.18 ± 0.29* | 59.1 ± 10.9*^○^ |
| Preeclamptic | SKA-31 | control | *3* | 4.51 ± 1.65 | 86.9 ± 0.1 |
| *, significant from normotensive control; ○significant from previous treatment interaction effect; *(blocker), assessed via two-way ANOVA with Šídák's *post hoc* multiple comparisons test; pEC50 and max dilation significance assessed via one-way ANOVA with Šídák's *post hoc* multiple comparisons test; *P*<0.05 taken as for all statistical tests. SKA-31, naphtho[1,2-d]thiazol-2-ylamine; TRAM-34, 1-(2-chlorophenyl)diphenyl)methyl]-1H-pyrazole. ‘*n*’ each from a different patient. See also Figure 4. | | | | | |

**Table S5.** **Antibody characteristics.**

| Antibody (Ab) | amino acid epitope / *research resource identifiers (*RRID) | species raised in | [supplied] / [working] | supplier / catalog, batch number/s | peptide available to Ab | homology | accession  number | specificity / characterization citation/s^b.^ |
| --- | --- | --- | --- | --- | --- | --- | --- | --- |
| 1.i. SK3 / KCNN3 / K_Ca_2.3 | human N’, aa 2-21, intracellular; RRID:AB_2040130 | rabbit | 0.6 mg/ml /1:100 | Alomone, APC-025, AN04 | yes | 100% to mouse, pig, rat | [Q9UGI6](http://www.uniprot.org/uniprot/Q9UGI6) | [^9^](#_ENREF_9)^,^[^34^](#_ENREF_34)  - *knock-out verified* |
| ii. peptide | DTSGHFHDSGVGDLDEDPKC | synthetic | 40 mg/1:10 | BLP-PC025 | to above, 1.i. | - as above. | [Q9UGI6](http://www.uniprot.org/uniprot/Q9UGI6) | - as above. |
|  |  |  |  |  |  |  |  |  |
| 2.i. SK4 / KCNN4 / IK1 / K_Ca_3.1 | rat C’ aa 350-363, intracellular; RRID:AB_2039959 | rabbit | 0.8 mg/ml /1:100 | Alomone, APC-064 | yes | 100% to human, mouse, pig | [Q9QYW1](http://www.uniprot.org/uniprot/Q9QYW1) | [^9^](#_ENREF_9)^,^[^10^](#_ENREF_10) |
| ii. peptide | RQVRLKHRKLREQV(C) | synthetic | 40 mg /1:10 | BLP-PC064 | to above, 2.i. | - as above. | [Q9QYW1](http://www.uniprot.org/uniprot/Q9QYW1) | - as above. |
|  |  |  |  |  |  |  |  |  |
| 3.i. BK_Ca_α / KCNMA1 / K_Ca_1.1 | murine C’, 1184-1200; RRID:AB_2040091 | rabbit | 0.6 mg/ml / 1:100 | Alomone APC-107; lots AN07, APC107, AN0825 | yes | 100% to rat; 16/17, bovine, chicken, dog, human | [Q08460](http://www.uniprot.org/uniprot/Q08460) | [^26^](#_ENREF_26)^,^[^35^](#_ENREF_35)  - *knock-out verified.* |
| ii. peptide | (C)STANRPNRPKSRESRDK intracellular loop near C’ | synthetic | 40 mg /1:10 | Alomone BLP-PC107 | to above, 3.i. | - as above. | [Q08460](http://www.uniprot.org/uniprot/Q08460) | - as above. |
|  |  |  |  |  |  |  |  |  |
| 4.i. BK_Ca_α / KCNMA1 / K_Ca_1.1 | rat 199-213; RRID:AB_10915895 | rabbit | 0.8 mg/ml / 1:100 | Alomone APC-151; lot APC151AN0525 | yes | 14/15 (~93%), human, mouse, rat | [Q62976](http://www.uniprot.org/uniprot/q62976) | [^36^](#_ENREF_36)^,^[^37^](#_ENREF_37) |
| ii. peptide | (C)DSSNPIES(S)QNFYKD,  1^st^ extracellular loop | synthetic | 40 mg / 1:10 | Alomone BLP-PC151 | to above, 4.i. | - as above. | [Q62976](http://www.uniprot.org/uniprot/q62976) | - as above. |
|  |  |  |  |  |  |  |  |  |
| 5. BK_Ca_β1 / slob1 / KCNMB1 | bovine 118-13 | rabbit | serum / 1:1000 | Merck, Garcia ^a.^ | serum only | - | - | [^26^](#_ENREF_26) |
|  |  |  |  |  |  |  |  |  |
| 6.i. BK_Ca_β1 / slob1 / KCNMB1 | rat 2-17; RRID:AB_2040095 | rabbit | 0.8 mg/ml / 1:100 | Alomone APC-036, lot APC036AN0502 | yes | 100% to dog, human, mouse, rabbit | [P97678](http://www.uniprot.org/uniprot/p97678) | [^36^](#_ENREF_36)^,^[^38^](#_ENREF_38); *noting limitations*[^39^](#_ENREF_39)^,^[^40^](#_ENREF_40) |
| ii. peptide | KKLVMAQKRGETRALC, intracellular near N’ | synthetic | 40 mg/ 1:10 | Alomone BLP-PC036 | to above, 6.i. | - as above. | [P97678](http://www.uniprot.org/uniprot/p97678) | - as above. |
|  |  |  |  |  |  |  |  |  |
| 7.i. caveolin-1 | human, N’; RRID:AB_2072042 | rabbit | 0.2 mg/ml / 1:100 | Santa Cruz, sc-894 | yes | to detect human, mouse, rat; not stated by manufacturer. | [Q03135](https://www.ebi.ac.uk/interpro/protein/reviewed/Q03135/) | [^11^](#_ENREF_11)^,^[^12^](#_ENREF_12) |
| ii. peptide | not stated by manufacturer | synthetic | 20 mg / 1:10 | Santa Cruz, sc-894P | to above, 7.1. | - as above. | [Q03135](https://www.ebi.ac.uk/interpro/protein/reviewed/Q03135/) | - as above |
|  |  |  |  |  |  |  |  |  |
| 8.i. caveolin 3 | mouse, N’; RRID:AB_637945 | goat | 0.2 mg/ml / 1:100 | Santa Cruz, sc-7665; lot C3106 | yes | to detect human, mouse, rat; not stated by manufacturer. | [P51637](https://www.ncbi.nlm.nih.gov/protein/P51637) | [^11^](#_ENREF_11)^,^[^12^](#_ENREF_12) |
| ii. peptide | not stated by manufacturer. | synthetic | 20 mg / 1:10 | Santa Cruz, sc-7665 P | to above, 8.i. | - as above. | [P51637](https://www.ncbi.nlm.nih.gov/protein/P51637) | - as above. |
| 9. eNOS | human, 1150-1200; RRID:AB_304967 | rabbit | 0.2-1 ml/mg / 1:100 | Abcam, ab5589 | yes | to detect human, mouse, rat. | [P29474](https://www.uniprot.org/uniprotkb/P29474/entry) | [^41^](#_ENREF_41) |
| 10. eNOS | human (pS1177) | mouse | 250 μg/ml / 1:500 | BD Transduction Labs  610297 lot#02128 | no | to detect human, bovine, dog, mouse, rat. | [P29474](https://www.uniprot.org/uniprotkb/P29474/entry) | [^11^](#_ENREF_11)^,^[^12^](#_ENREF_12) |
| 11. i. eNOS | Human C’;  RRID:AB_631423 | rabbit | 100μg/ml; 1:100 | Santa Cruz, sc-654 | yes | to detect human. | [P29474](https://www.uniprot.org/uniprotkb/P29474/entry) |  |
| ii. peptide | not stated by manufacturer. | synthetic | 20 mg / 1:10 | Santa Cruz, sc-654 P | to above, 11.i. | - as above. | [P29474](https://www.uniprot.org/uniprotkb/P29474/entry) |  |
| 12. donkey anti-rabbit 633 | rabbit IgG | rabbit | 2 mg/ml / 1:100 | Sigma-Merck SAB4600132, lots 20C1006 and 21C0928 | - | - | - | - |
|  |  |  |  |  |  |  |  |  |
| 13. donkey-anti-goat 555 | goat IgG | goat | 2 mg/ml / 1:100 | Sigma-Merck SAB4600059 | - | - | - | - |
|  |  |  |  |  |  |  |  |  |
| 14. donkey anti-goat 633 | goat IgG | goat | 2 mg/ml / 1:100 | Sigma-Merck, SAB4600128 | - | - | - | - |
| 15. 633 | mouse IgG | goat | 2 mg/ml / 1:100 | Invitrogen A21050 | - | - | - | - |

**a**. From Maria Garcia, Merck Research Laboratories, NJ, U.S.A. **b.** Includes characterization data, with comparison to tissue of known expression, and not only previous use. Of note, the three eNOS and two different BKα and BKβ antibodies had similar EC- and SM-selective labelling properties (**Figures 5** and **6,** respectively).

| **Table S6.** **Uterine radial artery endothelial caveolae density per µm vessel length.** | | | | | | | | | | | |
| --- | --- | --- | --- | --- | --- | --- | --- | --- | --- | --- | --- |
| (*n*=*4*, each) | **normotensive** | **preeclamptic** | | | **normotensive + pravastatin** | **preeclamptic + pravastatin** | | **normotensive + MβCD** | | **preeclamptic + MβCD** | |
| Lumenal  caveolae | 4.4 ± 0.3 | | 1.8 ± 0.1^○^ | 2.5 ± 0.1* | | | 3.2 ± 0.2* | | 2.8 ± 0.5* | | 3.1 ± 0.5 |
|  |  | |  |  | | |  | |  | |  |
| Lumenal submembranous caveolae | 8.5 ± 0.7 | | 4.2 ± 0.5^○^ | 5.9 ± 0.3* | | | 7.2 ± 0.7* | | 6.3 ± 0.8 | | 7.8 ± 1.0* |
|  |  | |  |  | | |  | |  | |  |
| Ablumenal  caveolae | 3.6 ± 0.2 | | 1.4 ± 0.2^○^ | 1.2 ± 0.1* | | | 1.5 ± 0.2 | | 2.5 ± 0.3*^#^ | | 2.8 ± 0.4*^#^ |
|  |  | |  |  | | |  | |  | |  |
| Ablumenal submembranous caveolae | 8.2 ± 0.8 | | 3.4 ± 0.5^○^ | 4.3 ± 0.4* | | | 4.0 ± 0.6 | | 8.3 ± 1.2^#^ | | - 1. ± 1.1*^#^ |

*Significance of pravastatin- or MβCD-treated PE or NT arteries *cf/.* untreated control; ^○^significance of untreated PE arteries *cf/.* untreated NT;
^#^ significance of MβCD-treated PE or NT arteries *cf/.* pravastatin-treated counterparts; *P*<0.05; Welch’s unpaired t-test. *n*, each from a different patient.

**Table S7.** **rt-qPCR Primers.**

| Gene | Primers | | Accession ID(s) |
| --- | --- | --- | --- |
| *CAV1* | Forward | 5’- GCGACCCTAAACACCTCAAC-3’ | NM_001172895.1; NM_001172896.2; NM_001753.5 |
|  | Reverse | 5’- ACAGCAAGCGGTAAAACCAG-3’ |  |
| *CAV2* | Forward | 5’- ACGACTCCTACAGCCACCAC-3’ | NM_001206747.2; NM_001233.5; NM_198212.3 |
|  | Reverse | 5’- CGTCCTACGCTCGTACACAA-3’ |  |
| *CAV3* | Forward | 5’-GAGGACATAGTCAAGGTGGATTT-3’ | NM_001234.5; NM_033337.3; |
|  | Reverse | 5’-GTACTTGGAGACAGTGAAGGTG-3’ |  |
| *CAVIN1* | Forward | 5’-AAGAAGCTGGAGGTCAACGA-3’ | NM_012232.6 |
|  | Reverse | 5’-CTCCGACTCTTTCAGCGATT-3’ |  |
| *CAVIN2* | Forward | 5’- AGCTGGGGACAAAGATCGTA-3’ | NM_004657.6 |
|  | Reverse | 5’- TCCTCTTCCTGGTCATTTGG-3’ |  |
| *GAPDH* | Forward | 5’- CATGGCCTTCCGTGTTCCTA- 3’ | X02231 |
|  | Reverse | 5’- TACTTGGCAGGTTTCTCCAGG-3’ |  |

**Supplemental References**

1. Brownfoot FC, Tong S, Hannan NJ, et al. Effects of pravastatin on human placenta, endothelium, and women with severe preeclampsia. *Hypertension*. 2015;**66**:687-97.

2. Brownfoot FC, Tong S, Hannan NJ, Hastie R, Cannon P, Kaitu’u-Lino TuJ. Effects of simvastatin, rosuvastatin and pravastatin on soluble fms-like tyrosine kinase 1 (sFlt-1) and soluble endoglin (sENG) secretion from human umbilical vein endothelial cells, primary trophoblast cells and placenta. *BMC Preg Child* 2016;**16**:117.

3. Absi M, Burnham MP, Weston AH, Harno E, Rogers M, Edwards G. Effects of methyl beta-cyclodextrin on EDHF responses in pig and rat arteries; association between SK_Ca_ channels and caveolin-rich domains. *Brit J Pharmacol* 2007;**151**:332-340.

4. Al-Brakati AY, Kamishima T, Dart C, Quayle JM. Caveolar disruption causes contraction of rat femoral arteries via reduced basal NO release and subsequent closure of BK_Ca_ channels. *PeerJ*. 2015;**2015**:e966-e966.

5 Brownfoot FC, Hannan N, Onda K, Tong S, Kaitu'u-Lino T. Soluble endoglin production is upregulated by oxysterols but not quenched by pravastatin in primary placental and endothelial cells. *Placenta*. 2014;**35**:724-731.

6. Chaiworapongsa T, Chaemsaithong P, Yeo L, Romero R. Pre-eclampsia part 1: current understanding of its pathophysiology. *Nat Rev Nephrol*. 2014;**10**:466-80.

7. Possomato-Vieira JS, Khalil RA. Mechanisms of endothelial dysfunction in hypertensive pregnancy and preeclampsia. *Adv Pharmacol* 2016;**77**:361-431.

8. Schwartz KS, Stanhewicz AE. Maternal microvascular dysfunction during and after preeclamptic pregnancy. *Compr Physiol*. 2024;**14**:5703-5727.

9. Sandow SL, Neylon CB, Chen MX, Garland CJ. Spatial separation of endothelial small- and intermediate-conductance calcium-activated potassium channels (K_Ca_) and connexins: possible relationship to vasodilator function? *J Anat*. 2006;**209**:689-98.

10. McNeish AJ, Sandow SL, Neylon CB, Chen MX, Dora KA, Garland CJ. Evidence for involvement of both IK_Ca_ and SK_Ca_ channels in hyperpolarizing responses of the rat middle cerebral artery. *Stroke* 2006;**37**:1277-1282.

11. Howitt L, Grayson TH, Morris MJ, Sandow SL, Murphy TV. Dietary obesity increases NO and inhibits BK_Ca_-mediated, endothelium-dependent dilation in rat cremaster muscle artery: association with caveolins and caveolae. *Am J Physiol*. 2012;**302**:H2426-2476.

12. Grayson TH, Chadha PS, Bertrand PP, et al. Increased caveolae density and caveolin-1 expression accompany impaired NO-mediated vasorelaxation in diet-induced obesity. *Histochem Cell Biol*. 2013;**139**:309-21.

13. Chadha PS, Lu L, Rikard-Bell M, et al. Endothelium-dependent vasodilation in human mesenteric artery is primarily mediated by myoendothelial gap junctions, IK_Ca_ and NO. *J Pharmacol Exp Ther*. 2011;**336**:701-708.

14. Senadheera S, Bertrand PP, Grayson TH, Leader L, Murphy TV, Sandow SL. Pregnancy-induced remodelling and enhanced endothelium-derived hyperpolarization-type vasodilator activity in rat uterine radial artery: TRPV4 channels, caveolae and myoendothelial gap junctions. *J Anat*. 2013;**223**:677-86.

15. Gillham JC, Myers JE, Baker PN, Taggart MJ. Regulation of endothelial-dependent delaxation in human systemic arteries by SK_Ca_ and IK_Ca_ channels. *Reprod Sci* 2007;**14**:43-50.

16. Dora KA, Gallagher NT, McNeish A, Garland CJ. Modulation of endothelial cell K_Ca_3.1 channels during EDHF signaling in mesenteric resistance arteries. *Circ Res*. 2008;**102**:1247-55.

17. Matoba T, Shimokawa H, Kubota H, et al. Hydrogen peroxide is an endothelium-derived hyperpolarizing factor in human mesenteric arteries. *Biochem Biophys Res Commun*. 2002;**290**:909-13.

18. Mauban JR, Wier WG. Essential role of EDHF in the initiation and maintenance of adrenergic vasomotion in rat mesenteric arteries. *Am J Physiol* 2004;**287**:H608-16.

19. Hilgers RH, Janssen GM, Fazzi GE, De Mey JG. Twenty-four-hour exposure to altered blood flow modifies endothelial Ca^2+-^activated K^+^ channels in rat mesenteric arteries. *J Pharmacol Exp Ther*. 2010;**333**:210-7.

20. Sankaranarayanan A, Raman G, Busch C, et al. Naphtho[1,2-d]thiazol-2-ylamine (SKA-31), a new activator of K_Ca_2 and K_Ca_3.1 potassium channels, potentiates the endothelium-derived hyperpolarizing factor response and lowers blood pressure. *Mol Pharmacol* 2009;**75**:281-95.

21. McGuire JJ, Dai J, Andrade-Gordon P, Triggle CR, Hollenberg MD. Proteinase-activated receptor-2 (PAR2): vascular effects of a PAR2-derived activating peptide via a receptor different than PAR2. *J Pharmacol Exp Ther*. 2002;**303**:985-92.

22. Smith WL, Lecomte M, Laneuville O, Lecomte M, Breuer DK, DeWitt DL. Differential inhibition of human prostaglandin endoperoxidase H synthasees-I and -2 by aspiring and other nonsteroidal antiinflammatory drugs. *Eur J Med Chem*. 1995;**30**:417s-427s.

23. Weston AH, Porter EL, Harno E, Edwards G. Impairment of endothelial SK_Ca_ channels and of downstream hyperpolarizing pathways in mesenteric arteries from spontaneously hypertensive rats. *Br J Pharmacol*. 2010;**160**:836-43.

24. Eichler I, Wibawa J, Grgic I, et al. Selective blockade of endothelial Ca^2+^-activated small- and intermediate-conductance K^+^-channels suppresses EDHF-mediated vasodilation. *Br J Pharmacol*. 2003;**138**:594-601.

25. Zhou Y, Lingle CJ. Paxilline inhibits BK channels by an almost exclusively closed-channel block mechanism. *J Gen Physiol*. 2014;**144**:415-40.

26. Howitt L, Sandow SL, Grayson TH, Ellis ZE, Morris MJ, Murphy TV. Differential effects of diet-induced obesity on BK_Ca_β1-subunit expression and function in rat skeletal muscle arterioles and small cerebral arteries. *Am J Physiol*. 2011;**301**:H29-40.

27. Costantine MM, West H, Wisner KL, et al. A randomized pilot clinical trial of pravastatin versus placebo in pregnant patients at high risk of preeclampsia. *Am J Obstet Gynecol* 2021;**225**:666.e1-666.e15.

28. Mione MC, Cavanagh JFR, Burnstock G. Uptake of 5-hydroxydopamine into non-sympathetic nerves of guinea-pig uterine artery in late pregnancy. *J Neurocytol* 1993;**22**:164-175.

29. Sandow SL. Factors, fiction and endothelium-derived hyperpolarizing factor. *Clin Exp Pharmacol Physiol* 2004;**31**:563-70.

30. Sandow SL, Haddock RE, Hill CE, et al. What's where and why at a vascular myoendothelial microdomain signalling complex. *Clin Exp Pharmacol Physiol* 2009;**36**:67-76.

31. Sandow SL, Senadheera S, Bertrand PP, Murphy TV, Tare M. Myoendothelial contacts, gap junctions, and microdomains: anatomical links to function? *Microcirculation*. 2012;**19**:403-15.

32. Sandow SL, Senadheera S, Grayson TH, Welsh DG, Murphy TV. Calcium and endothelium-mediated vasodilator signaling. *Adv Exp Med Biol*. 2012;**740**:811-31.

33. Félétou M. Endothelium-dependent hyperpolarization and endothelial dysfunction. *J Cardiovasc Pharmacol* 2016;**67**:373-87.

34. Kim S, Ma L, Jensen KL, Kim MM, Bond CT, Adelman JP, Yu CR. Paradoxical contribution of SK3 and GIRK channels to the activation of mouse vomeronasal organ. *Nat Neurosci*. 2012;**15**:1236-44.

35. Hei H, Gao J, Dong J, Tao J, Tian L, Pan W, Wang H, Zhang X. BK knockout by TALEN-mediated gene targeting in osteoblasts: KCNMA1 determines the proliferation and differentiation of osteoblasts. *Mol Cells*. 2016;**39**:530-5.

36. Balderas E, Torres NS, Rosa-Garrido M, et al. MitoBK_Ca_ channel is functionally associated with its regulatory β1 subunit in cardiac mitochondria. *J Physiol*. 2019;**597**:3817-32.

37. Lu M, Li JR, Alvarez-Lugo L, et al. Lipopolysaccharide stimulates BK channel activity in bladder umbrella cells. *Am J Physiol* 2018;**314**:C643-53.

38. Shi L, Liu X, Li N, Liu B, Liu Y. Aging decreases the contribution of MaxiK channel in regulating vascular tone in mesenteric artery by unparallel downregulation of α- and β1-subunit expression. *Mech Ageing Dev*. 2013;**134**:416-25.

39. Sandow SL, Grayson TH. Limits of isolation and culture: intact vascular endothelium and BK_Ca_. *Am J Physiol*. 2009;**297**:H1-7.

40. Bhattarai Y, Fernandes R, Kadrofske MM, Lockwood LR, Galligan JJ, Xu H. Western blot analysis of BK channel β1-subunit expression should be interpreted cautiously when using commercially available antibodies. *Physiol Rep*. 2014;**2**: e12189.

41. Zhang JH, Kawashima S, Yokoyama M, Huang P, Hill CE. Increased eNOS accounts for changes in connexin expression in renal arterioles during diabetes. *Anat Rec* 2006;**288A**:1000-08.
